# Transcriptomics and metabolomics analyses reveal pollen abortion mechanism in alfalfa early stage male sterile lines

**DOI:** 10.3389/fpls.2024.1464747

**Published:** 2024-12-17

**Authors:** Huicai Cai, Shuhe Zhang, Weijie Yu, Xue Jia, Lan Yu, Bo Xu, Yingzhe Wang

**Affiliations:** ^1^ Jilin Provincial Key Laboratory of Tree and Grass Genetics and Breeding, College of Forestry and Grassland Science, Jilin Agricultural University, Changchun, China; ^2^ Institute of Agricultural Biotechnology, Jilin Academy of Agricultural Sciences (JAAS), Changchun, China

**Keywords:** alfalfa, cytoplasmic male sterile lines, transcriptome sequencing, non-targeted metabolome sequencing, anthers

## Abstract

Alfalfa (*Medicago sativa* L.), a prominent perennial forage in the legume family, is widely cultivated across Europe and America. Given its substantial economic value for livestock, breeding efforts have focused on developing high-yield and high-quality varieties since the discovery of CMS lines. However, progress is restricted by the limitations of existing CMS lines, necessitating the development of new lines and study of the molecular mechanisms underlying pollen abortion. This study investigates early-stage anther development in cytoplasmic male sterile (CMS) alfalfa lines (MSJN1A) in relation to the isotypic maintainer line (MSJN1B). Histological analyses revealed abnormal degradation of tapetal cells post-meiosis in the CMS line. Notably, during the early mononuclear stage, the central vacuoles in the microspores were absent, leading to evident pollen abortion. These findings suggest that pollen abortion in the CMS line is associated with the delayed disintegration of the tapetum and structural anomalies in microspore vacuoles. Non-targeted metabolome sequencing revealed 401 and 405 metabolites at late tetrad and early mononuclear stages of alfalfa, respectively. Among these, 39 metabolites were consistently upregulated, whereas 85 metabolites were downregulated. Differential analysis revealed 45 and 37 unique metabolites at each respective stage. These metabolites were primarily featured in pathways related to energy, phenylpropane, sucrose and starch, and fatty acid metabolism. Integrated analysis demonstrated that differentially expressed genes and differential metabolites were co-enriched in these pathways. Additionally, quantitative real-time PCR and physiological index analysis confirmed downregulation of key genes involved in anther development, illustrating that changes in upstream gene regulation could significantly impact downstream metabolite levels, ultimately influencing pollen fertility. Pollen abortion is related to abnormal phenylpropane metabolism, fatty acid metabolism and starch and sucrose pathway, which provides reference for further research on the causes of pollen abortion of alfalfa.

## 1 Introduction

Alfalfa (*Medicago sativa L.*), a prominent perennial forage in the legume family, is widely cultivated across Europe and America. Given its substantial economic value for livestock, breeding efforts have focused on developing high-yield and high-quality varieties since the discovery of CMS lines. However, progress is restricted by the limitations of existing CMS lines, necessitating the development of new lines and study of the molecular mechanisms underlying pollen abortion.

Pollen development is a complex process involving multiple stages, including expansion and differentiation of flower buds, elongation of filaments, and transformation of stamen primordia into cystic anthers (Dong et al., 2013). As pollen mother cell mitosis progresses to a certain stage, early division of various anther cell structures occurs. During this phase, the tapetum layer expands and begins to accumulate substances, leading to abundant production of callose, ustenite, and starch granules. Subsequently, following meiosis of pollen mother cells, tapetal cells undergo programmed cell death during which complex macromolecules, such as sugars, lipids, and amino acids, are broken down into smaller molecules and transported to developing spores (Tang et al., 2018). Subsequently, long chain fatty acids, polysaccharides, and oxidized aromatic ring derivatives converge to synthesize sporopollenin—the crucial raw material for the construction of the anther cuticle and external pollen wall (Shi et al., 2015). The anther cuticle and pollen outer wall serve as a protective barrier for the spore body, shielding it from environmental disturbances and enabling its continued development in a relatively stable environment (Morant et al., 2003). As the anther sac expands, tapetum degradation ceases, allowing microsporocytes to enlarge and gradually disperse uniformly within the anther sac. During the binuclear stage, vacuoles within the pollen granulocytes progressively increase in size and nuclei migrate toward one side of the cell, culminating in the formation of a tri-cellular male gametophyte or a mature pollen grain (Yuan et al., 2022). Ultimately, the anther shell fractures, resulting in the formation of four fissures through which pollen grains are released into the external environment (Kretschmer et al., 2017). Recent findings indicate that the failure of pollen formation in CMS lines is linked to abnormal degradation of tapetal cells during the meiosis of pollen mother cells (Yang et al., 2019a; Chen et al., 2021). While the molecular mechanisms underlying male sterility in various plant species have been extensively documented, investigations into these processes in alfalfa are still in their nascent stages (Wang et al., 2021; Zhou et al., 2022). These investigations have yielded valuable insights into male sterility in alfalfa; however, mechanisms underlying pollen defeat vary across different alfalfa sterile lines. Therefore, further research is required to strengthen and deepen our understanding of the molecular regulatory mechanisms underlying pollen sterility in alfalfa species.

To elucidate the mechanism of pollen abortion in alfalfa, we utilized high-throughput sequencing technology to sequence alfalfa transcriptome based on morphological observations and conducted untargeted metabolomic analysis of early anther metabolites using liquid chromatography-mass spectrometry (LC-MS) (**Supplementary Figure 1**). A comprehensive library of alfalfa anther transcriptomes was established. Additionally, we identified and analyzed differentially expressed genes (DEGs) in sterile and maintainer lines during early anther development through integrative analyses of transcriptomic and metabolomic data. These efforts have provided crucial insights into the molecular regulatory mechanisms underlying pollen abortion in alfalfa.

## 2 Materials and methods

### 2.1 Acquisition of experimental materials

Using the Gongnong No. 3 variety of Medicago sativa as the starting material, we performed space-induced mutagenesis breeding to screen for CMS material MSJN1-A. The MSJN1-A line was developed using Gongnong No. 3 as the test parent and the maintainer line MSJN1B (Zhongmu No. 1) as the hybrid parent, followed by four generations of backcrossing. MSJN1-A was then crossed with the restorer line Longmu 803 to produce F1 seeds, and subsequent self-pollination of the F1 generation yielded F2 segregating progeny. All materials were provided by Laboratory 303 of the College of Forestry and Grassland, Jilin Agricultural University. In 2022, the MSJN1A and MSJN1B materials were planted in the Grass Science Experimental Field of Jilin Agricultural University in Changchun, Jilin Province, China (125°24′15″E, 43°48′47″N, altitude 203 m).

In the experimental field, during the full flowering stage, 10 groups of single plants at the same growth stage, including both sterile lines and maintainer lines, were selected. Temporary pollen slices were observed to select 3 groups of single plants. Based on the length of flower buds, staminate flower buds at different developmental stages were collected. Petals and florets were removed with tweezers, leaving only the anthers. Some anthers were used for semi-thin sectioning (**Supplementary Figure 2**), while the remaining samples were rapidly frozen in liquid nitrogen and stored at -80°C for subsequent experiments.

### 2.2 Cytological observation

Following the method of Huang et al (Lemercier et al., 2017; Huang et al., 2023), anther resin blocks were sectioned using an ultramicrotome (Leica UC7, Japan). Anther tissue was stained using toluidine blue and observed under a microscope.

### 2.3 RNA extraction, cDNA library construction, transcriptome sequencing, and non-targeted metabolome sequencing

Our laboratory tentatively confirmed that anther abortion in alfalfa begins at the late tetrad stage, with significant abortion occurring at the mononuclear stage. Therefore, we selected late tetrads and mononuclear early anthers for transcriptome and metabolome sequencing. Three biological replicates were prepared for each sample. RNA was extracted using a plant RNA extraction kit, and RNA purity and concentration were measured using a micro-UV spectrophotometer. Sequencing libraries were generated using NEBNext^®^Ultra™ RNA library prep kit for Illumina^®^ (NEB, USA) following manufacturer’s recommendations and index codes were added to attribute sequences to each sample. Sample sequencing and splicing were performed on Illumina HiSeq high-throughput sequencing platform at BioMarker Technologies (Beijing, China).

LC-MS was used to assay metabolites at early anther stages. It consists of an ultra-high-performance liquid-phase (Waters, Acquity I-Class PLUS, American) tandem high-resolution mass spectrometer (Waters, Xevo G2-XS QTof, American). Chromatographic columns were purchased from Waters Corporation (Acquity UPLC HSS T3, American) (1.8 um 2.1 × 100 mm). Metabolite detection on LC-MS was performed by BioMarker Technologies (Beijing, China).

### 2.4 Transcriptome assembly and functional gene annotation

This study utilized the Zhongmu No.1 reference genome for reference-based transcriptome assembly and gene annotation. Quality-controlled clean reads were aligned to the Zhongmu No.1 genome using HISAT2 (v2.2.1), followed by sorting and indexing with SAMtools (v1.10). Transcript assembly was then performed with StringTie (v2.1.4) to reconstruct the complete transcriptome structure. The assembled transcripts were annotated using the annotation file (GTF format) from the Zhongmu No.1 reference genome (Sun et al., 2023). Functional enrichment analysis was carried out using the KEGG (Kyoto Encyclopedia of Genes and Genomes) and GO (Gene Ontology) databases to elucidate the biological roles of these genes in the anther development of alfalfa.

### 2.5 Differential expression analysis and enrichment analysis of DEGs

Differential expression analysis of two groups was performed using the DESeq R package (1.10.1). DESeq provide statistical routines to determine differential expression in digital gene expression data using a model based on the negative binomial distribution. The resulting P values were adjusted using the Benjamini and Hochberg’s approach to control the false discovery rate. Genes with an adjusted P-value <0.05 (as determined by DESeq) were assigned as DEGs. GO (Anders and Huber, 2010; Bu et al., 2021) and KEGG pathway enrichment analyses (Ashburner et al., 2000) of the DEGs were implemented using the topGO R package and KOBAS (v3.0) software (Kanehisa and Goto, 2000).

### 2.6 Quantitative real-time (qRT)-PCR validation

QRT-PCR assays were conducted using LightCycler^®^ 96 (LightCycler^®^, Switzerland) following the protocol provided with the TB Green^®^ premix Ex Taq™ II (Takara). The β-actin gene was used as the internal reference. A negative control was established for each gene to ensure specificity, and three biological replicates were incorporated into all experimental designs. The relative expression levels of the target genes were quantified utilizing the 2^−ΔΔCT^ method (Bubner and Baldwin, 2004).

### 2.7 Metabolites screening and functional annotation for metabolome sequencing

The raw data collected from MassLynx V4.2 were processed using Progenesis QI software for peak extraction and alignment and other data processing operations. The raw data were identified based on the METLIN database of Progenesis QI software and BioMarker Technologies (Beijing, China) library. Theoretical fragments were identified. The mass number deviation was within 100 ppm.

### 2.8 Differential expression analysis and enrichment analysis of differential metabolites

PCA and partial least-squares discrimination analysis were used to analyze the degree of separation between groups. The DM standards of statistics were |log2FC|>1, *P*<0.05, and VIP>1. The annotation of DMs was done using the KOBAS (v3.0) software.

### 2.9 Analysis of physiological indicators

Reagents to assess physiological indicators were obtained from Nanjing Jiancheng Bioengineering Institute. Anthers from various developmental stages (each weighing 0.1 g) were homogenized, centrifuged, and the supernatant was analyzed for specific indices. To ensure accuracy, three biological replicates were prepared for each sample, with statistical significance assessed using one-way ANOVA. To estimate soluble sugars and starch, absorbance was measured at 625 nm through anthranone colorimetry using a plant soluble sugar determination kit (item no. A145-1-1) (Morris, 1948). Soluble proteins were quantified using Bradford’s method (Kielkopf et al., 2020). Proline content was determined using the PRO assay kit (item no. A107-1-1) by measuring absorbance at 520 nm using the acid ninhydrin method (Bates et al., 1973). To determine superoxide dismutase (SOD) activity, 0.1 g tissue sample was homogenized in 1 mL of 50 mM phosphate buffer, centrifuged at 15,000 x g at 4°C for 15 min, and the supernatant containing SOD was collected. To prepare the reaction mixture, 880 µL of phosphate buffer, 100 µL of guaiacol (0.1% final concentration), and 20 µL of hydrogen peroxide (3 mM final concentration) were mixed with 50 µL of enzyme extract. Absorbance was measured at 470 nm using the guaiacol method (Hibberd and Weber, 2012). Malondialdehyde (MDA) content was determined using the plant MDA determination kit (item no. A003-3-1) employing the thiobarbituric acid colorimetric method by measuring absorbance at 532 nm (Heath and Packer, 1968). Samples were centrifuged in Verstai M22R (ESCO, Singapore). Absorbance was measured at 721 nm in a visible spectrophotometer (Shanghai Jing Hua Scientific Instruments Co. Ltd., Shanghai, China) and UV1050 spectrophotometer (Shanghai Tianmei Scientific Instruments Co. Ltd, Shanghai, China).

### 2.10 Transcriptome and metabolome co-analysis

First, transcriptomic and metabolomic data are normalized to eliminate technical biases. Correlation Analysis: Spearman or Pearson correlation analysis is performed to assess the relationships between gene expression levels and metabolite abundances. Co-expression Network Analysis: A co-expression network is constructed to integrate transcriptomic and metabolomic data, allowing for the identification of potential regulatory relationships. The Weighted Gene Co-expression Network Analysis (WGCNA) tool is employed to construct co-expression networks between genes and metabolites, facilitating the exploration of key gene-metabolite modules to identify potential regulatory interactions. Pathway Enrichment Analysis: Pathway enrichment analysis of differentially expressed genes (DEGs) and significantly altered metabolites is conducted using KEGG, Gene Ontology (GO), and MetaboAnalyst tools. Visualization: Network and pathway maps are visualized using Cytoscape software, generating heatmaps and network diagrams to illustrate the relationships between genes and metabolites.

## 3 Result

### 3.1 Abnormal chorion degradation and microspore disappearance result in pollen abortion in sterile lines

**Figure 1:**
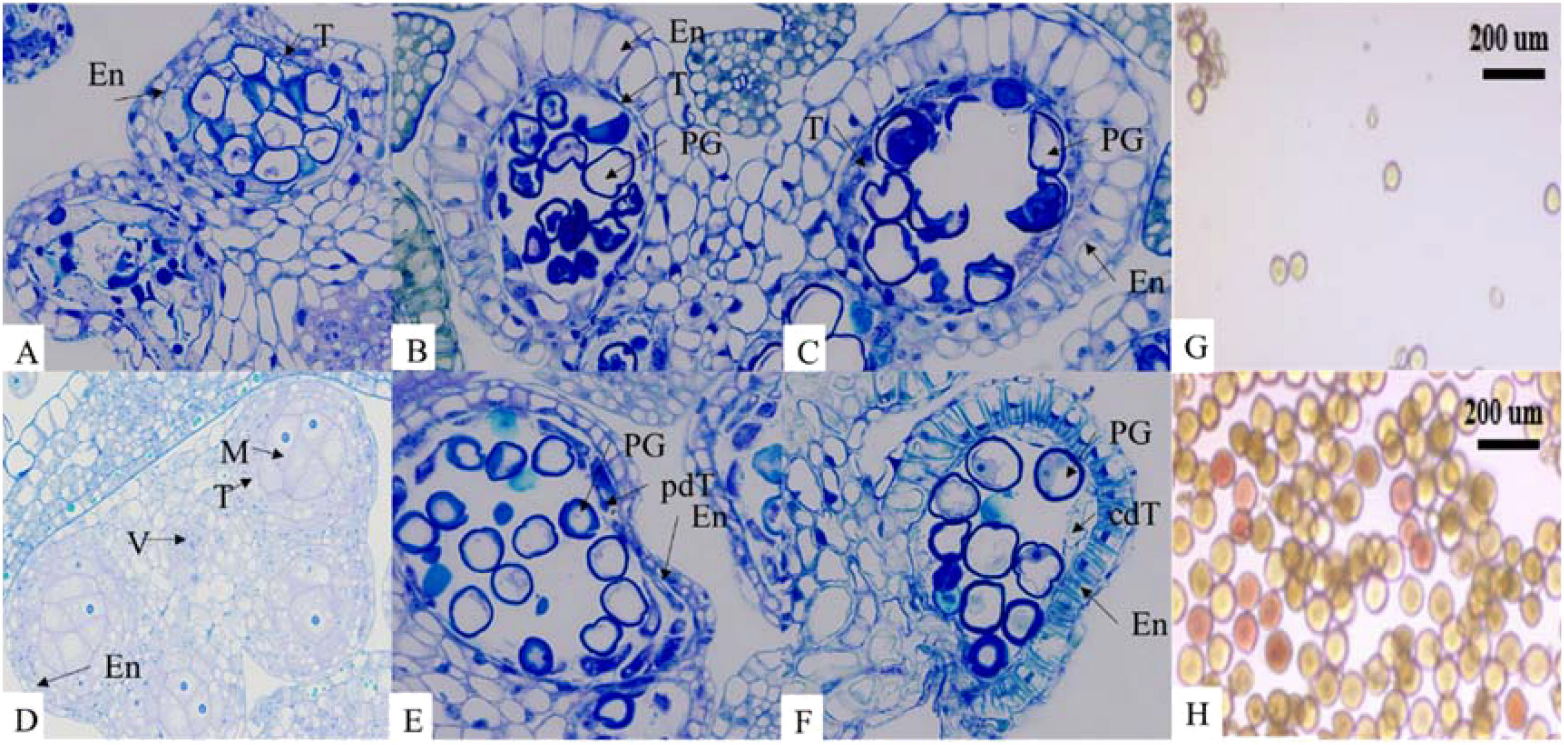
T: tapetum; M: microspore; V: vascular region; PG: pollen grain; En: endothecium; pdT: partially degraded tapetum; cdT: completely degraded tapetum. Sterile lines: **(A-C)** Maintainer lines: **(D-F)**. **(A, D)**: early anther tetrads; **(B, E)**: late anther tetrads; **(C, F)**: early mononuclear stage. Scale bar = 10 µm for all stages. **(G)**: TTC staining of pollen grains in sterile lines; **(H)**: TTC staining of pollen grains in maintainer lines. Scale bar = 200 µm for pollen grain staining. The microscope magnification is 400x.

**Figure 2:**
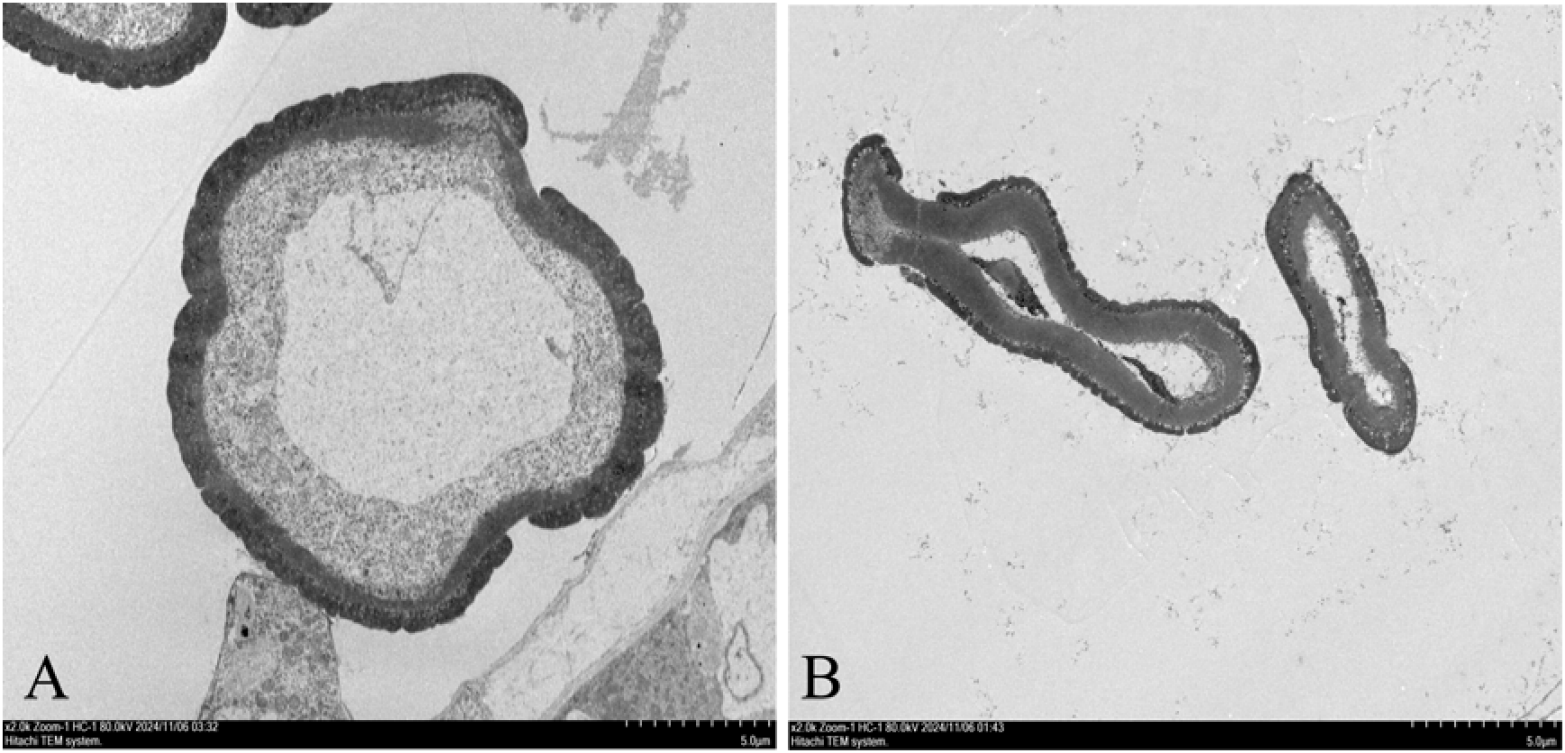
Electron microscopic image of microspore vacuoles at anther tetrad stage. **(A)**: MSJN1B. **(B)**: MSJN1A. Scale bar = 5.0 µm.

At the early anther tetrad stage, the two lineages had similar structures in the anther capsule and the cells of the tomentum layer were stained darker. The pollen mother cells in the anther capsule of the maintenance line were divided into microspores through meiosis and the pollen grains were rounded and evenly distributed in the anther capsule. The nuclei were on one side of the cells, the vesicles became larger, and the fleece layer was degraded during this process. Contrarily, there was no obvious degradation of the felted layer in the anther capsule of the sterile tetrad, and the pollen grains were irregular in shape. As the developmental stage proceeded, the felted layer of the sterile anther was still clearly visible, the pollen grains were haphazardly distributed in the anther capsule, and the pollen grains were dried up and partially degraded, showing abnormal nutrient accumulation (**Figure 1**). These observations were similar to other CMS plants with programmed cell death abnormalities in the tapetal cells (Liu et al., 2018; Du et al., 2019; Zhang et al., 2023). After dispersal, only few pollen grains from sterile lines were observed and pollen vigor was lacking. The abortion of anthers in sterile lines was found to be associated with the delayed degradation of the fleece layer and the non-formation of the central large vesicle (**Figure 2**).

### 3.2 Transcriptome sequencing of alfalfa

The data was filtered and screened to obtain high-quality clean reads for subsequent analysis. Thus, 5.94 Gb of clean reads were obtained from each sample. The GC content of reads in the samples (%) and the percentage of Q30 bases exceeded 92.19% for all samples. The length of the assembled gene fragments was analyzed (**Supplementary Table 1**). The proportion of 1000~2000 bp transcripts was found to be the highest (23.43%). The proportion of 200~300 bp in a single gene was the highest (33.60%).

### 3.3 Screening of DEGs

**Figure 3:**
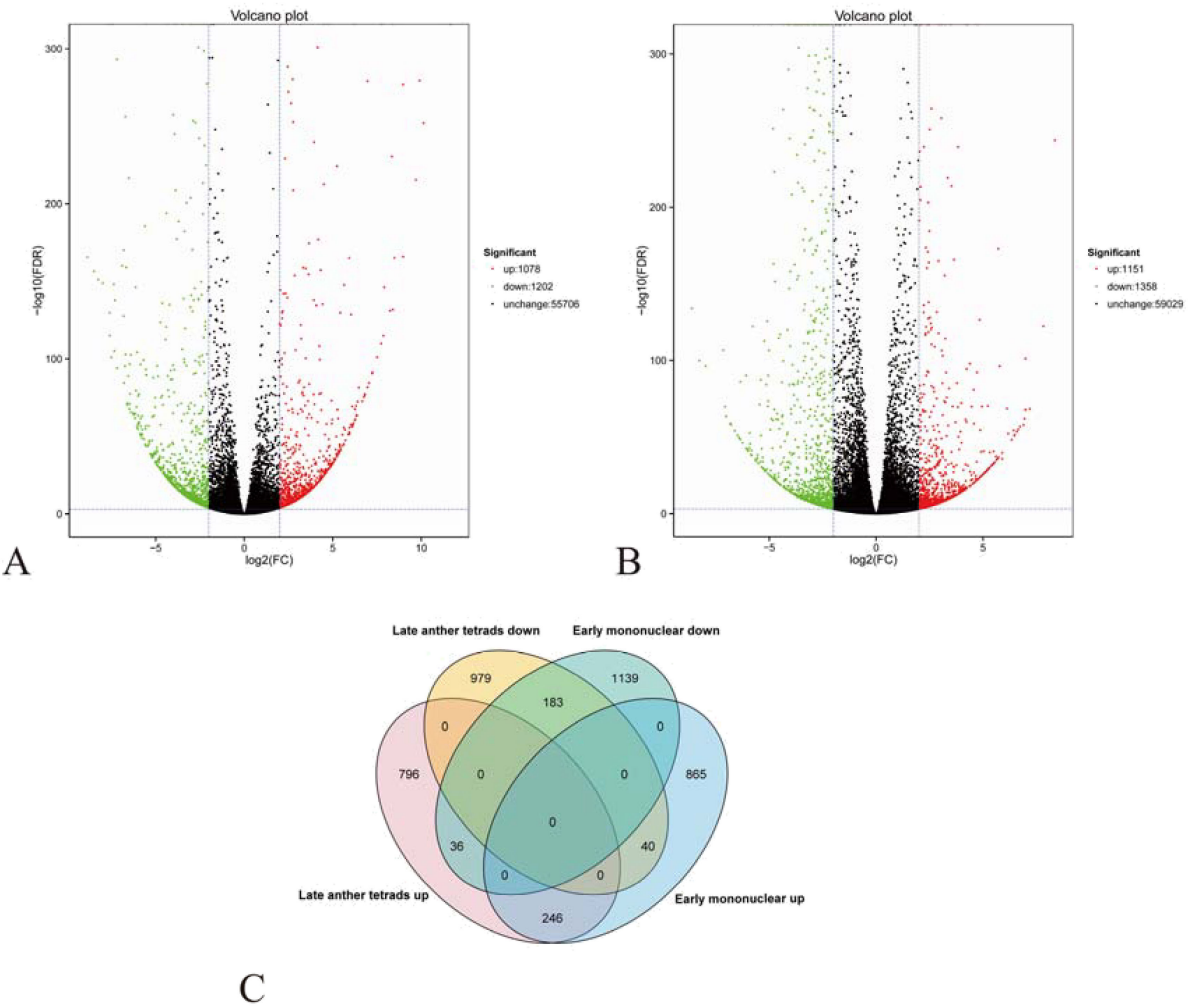
**(A, B)**: Volcano maps of differentially expressed genes between samples; **(C)**: Co-up-regulated and co-down-regulated DEGs in late tetrad and early mononuclear phases.

DESeq2 software was used to screen the sequencing results (|log2FC|>2, FDR<0.05). A total of 57,986 and 61,538 genes were detected at late tetrad and early mononuclear stages, respectively. Of these genes, 2,280 and 2,509 DEGs were detected at late tetrad and early mononuclear stages, respectively. The sterile line contained 1,078 and 1,358 upregulated genes at late tetrad and early mononuclear stages, respectively and 1,151 and 1,358 downregulated genes at late tetrad and early mononuclear stages, respectively. We compared DEGs obtained in our study with various databases (**Supplementary Table 2**). A total of 1,677 and 1,761 annotated genes were obtained in each period, respectively. For detailed annotation of TOP genes (|log2FC|>4), see **Supplementary Tables 3** and **4**. The Nr database is the most annotated one, accounting for 99.07% and 98.07% of DEGs, respectively. In addition, the male sterile line contained 1,078 and 1,151 upregulated genes at late tetrad and early mononuclear stages, respectively and 1,202 and 1,358 downregulated genes at late tetrad and early mononuclear stages, respectively (**Figure 3**). The function of Nr was annotated by Diamond software (vo.8.22), and e-value was set as 1e−5. Nr function was annotated in the single gene. Among them, 1,091 and 1,134 single gene sequences at tetrad and early mononuclear stages, respectively, were highly homologous to those found in the Leguminosae family, accounting for more than 95% of the total gene sequences (**Supplementary Figure 4**).

### 3.4 GO function annotation of DEGs

**Figure 4:**
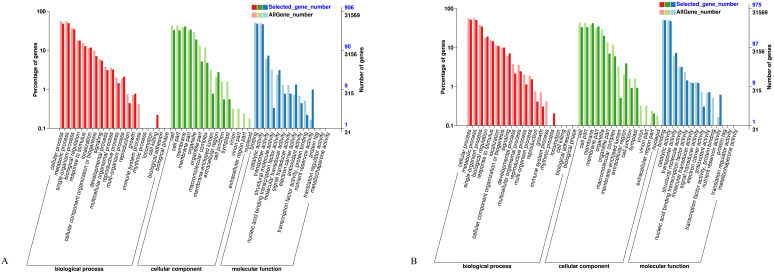
GO annotation statistics of differentially expressed genes. **(A)**: Late tetrad; **(B)**: Early mononuclear stage.

To explore the biological functions of DEGs and the metabolic pathways involved in the development of alfalfa anthers, GO functional enrichment analysis was performed on DEGs at late anther tetrad and early mononuclear stages. As shown in (**Figure 4**), there were three groups, namely, biological processes, cell components, and molecular functions. The study of DEGs was annotated into 49 terms in the GO classification, among which there were 20 subclasses of biological processes, focusing on cellular processes, metabolic processes, single-organism processes, and biological regulation. There were 15 subclasses of cell components, which were concentrated in cells, cell parts, membranes, membrane parts, and organelles. There were 14 subclasses of molecular functions, focusing on binding, catalysis, transport, and molecular structure.

### 3.5 KEGG function annotation of DEGs

**Figure 5:**
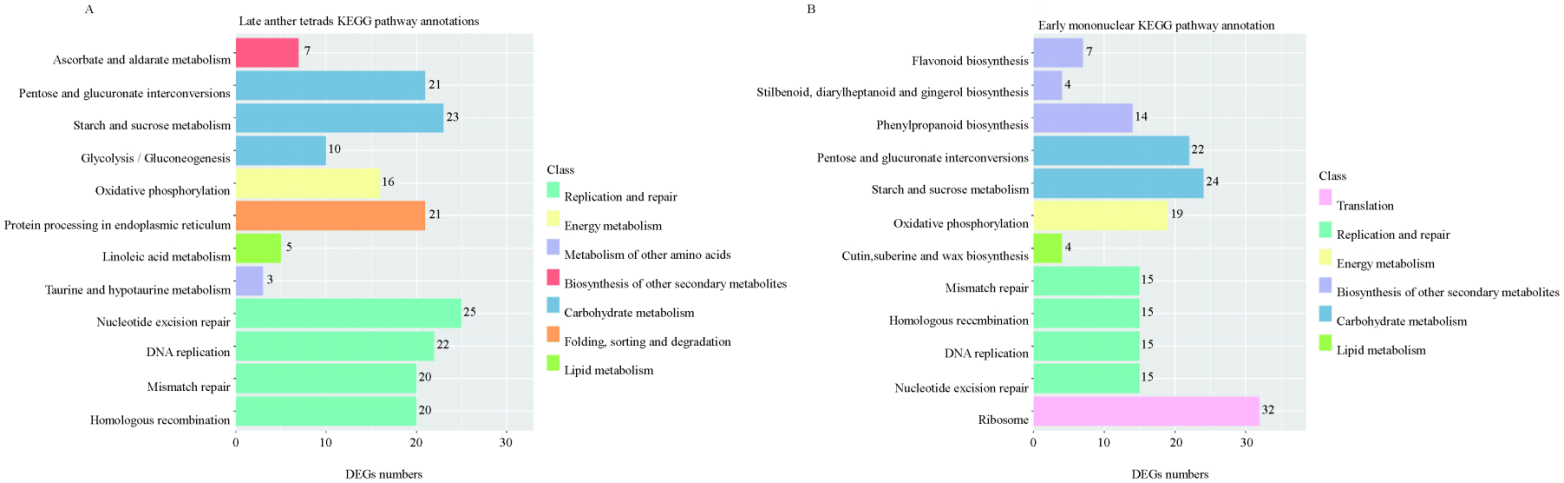
Partial KEGG analysis of DEGs. **(A)**: shows the KEGG pathway annotation of DEGs in late anther tetrads; **(B)**: the KEGG pathway annotation of DEGs in early mononuclear.

The DEGs (|log2FC|>4) with significant differences between the late tetrad and early mononuclear whole-genome databases were compared with Zhongmu No. 1 whole-genome databases. The corresponding functional annotations were made and clustering was performed. The comparative findings are shown in **Supplementary Tables 3** and **4**. According to KEGG pathway classification and statistical analysis, DEGs involved more than 20 pathways in five categories: cell process, environmental information processing, gene information processing, and metabolism. Further, DEGs involved in anther fertility were significantly enriched in the following metabolic pathways: biosynthesis of secondary metabolites, carbohydrate metabolism, energy metabolism, folding, sorting and degradation, lipid metabolism, metabolism of other amino acids, replication and restoration, etc. (**Figure 5**). The results showed that lipid, carbohydrate, and energy metabolism, nucleic acid mismatch repair, and other metabolic pathways occurring in the anthers of sterile lines were abnormal.

### 3.6 Anther development related transcription factor analysis

**Figure 6:**
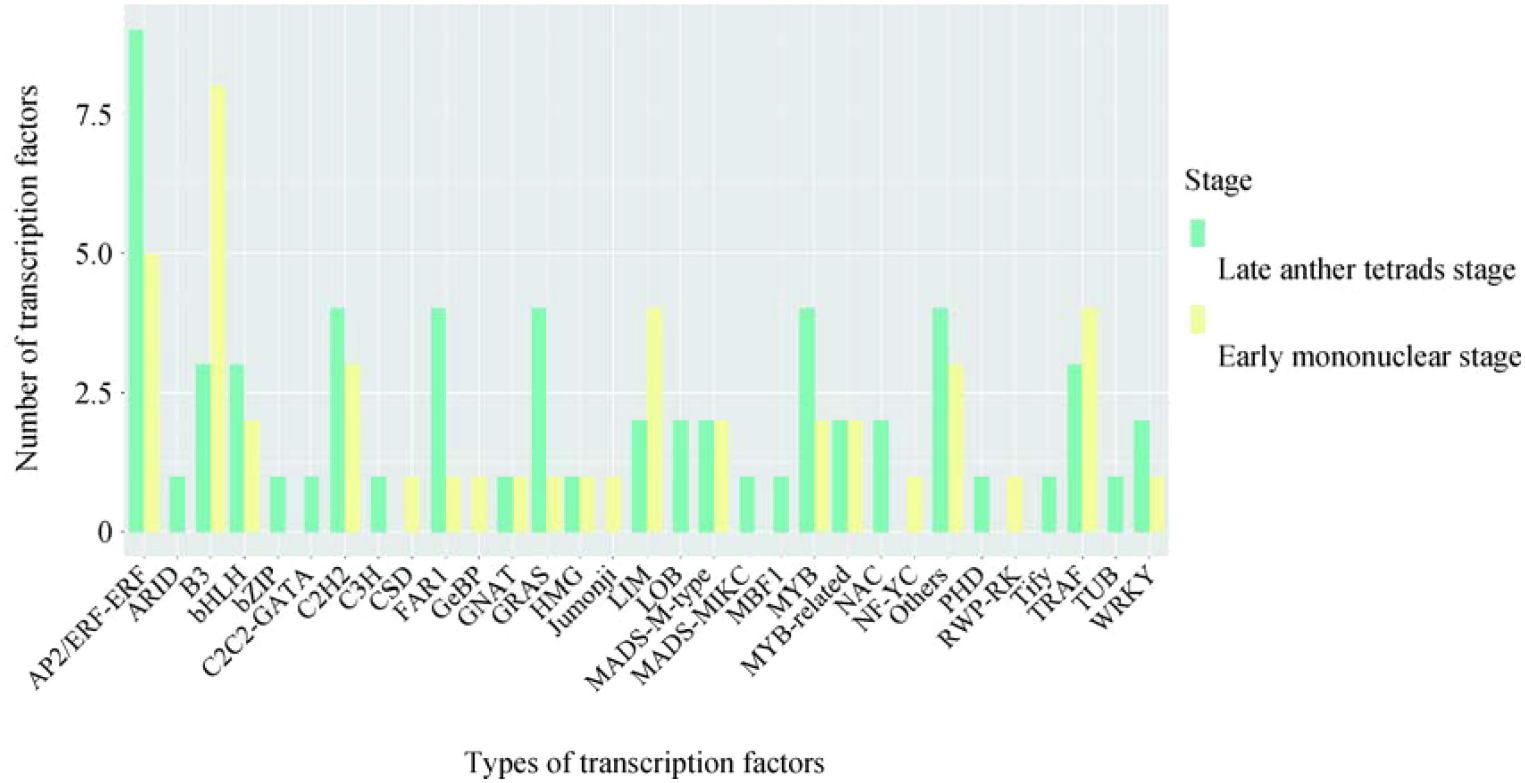
Anther transcription factor family analysis.

DEGs (106) in the obtained Unigene were annotated as transcription factors and transcriptional regulators, among which AP2/ERF-ERF (14), B3 (11), others (7), TRAF (7), etc. were the most annotated (**Figure 6**). It contains several families involved in regulating key metabolism during pollen development, such as MYB 35(c91687.graph_c0) and PHD finger protein male sterility 1 (c89108.graph_c0).

### 3.7 Metabolome sequencing of alfalfa

**Figure 7:**
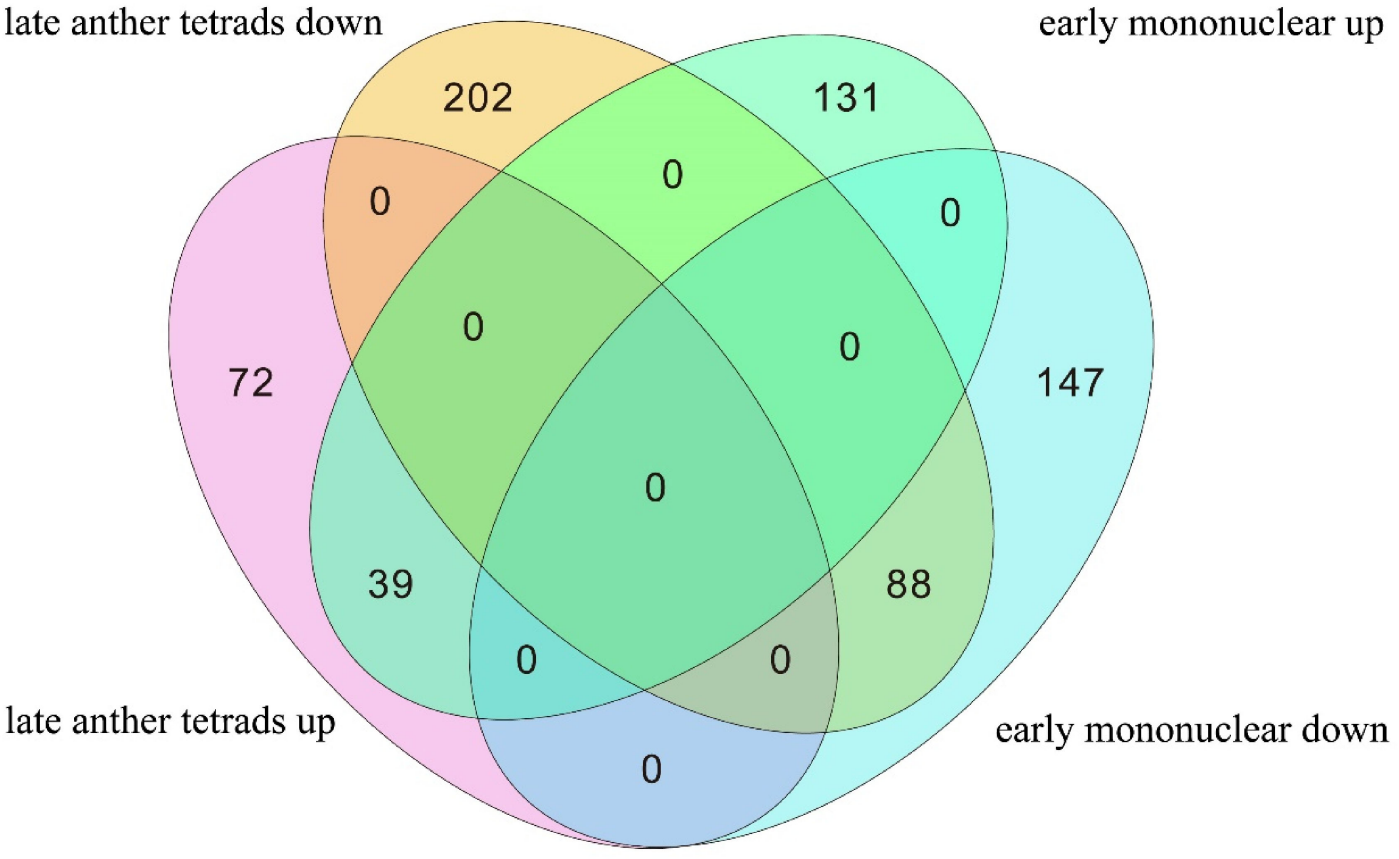
Statistics of DMs in early anther development.

Based on ultrahigh performance liquid chromatography tandem mass spectrometry (UPLC-MS/MS) detection platform, METLIN online database, and BioMarker Technologies (Beijing, China) library, data were collected from the pre-anther samples of two lines of alfalfa. A total of 908 metabolites from 24 samples were analyzed. The chromatographic results showed (**Supplementary Figure 4**) that the ion samples of the two groups were similar during the same period, indicating that metabolites between the two lines were generally the same. The PCA analysis clearly demonstrates significant differences in metabolite levels between the Maintainer line and the Male sterile line (**Supplementary Figure 5**). At the two developmental stages, 401 and 405 metabolites were identified, respectively, covering a broad spectrum of compounds, including sugars, organic acids, lipids, amino acids, carotenoids, and more, as shown in (**Figure 7**). Notably, during the early stage of anther development, 39 metabolites were consistently upregulated, while 88 metabolites were consistently downregulated, with no changes observed in their expression trends. These differentially expressed metabolites could be key factors contributing to pollen abortion.

### 3.8 Metabolite screening of alfalfa

DMs were selected based on these criteria: VIP>1, *P*<0.05, |log2FC |>2 (**Supplementary Tables 5**, **6**). A total of 45 and 37 different metabolites were identified at the late tetrad and early mononuclear stages, respectively. DMs mainly include carbohydrates, amino acids, flavonoids, phenylpropane, fatty acids, ribose, and other substances.

### 3.9 KEGG function annotation of DMs

**Figure 8:**
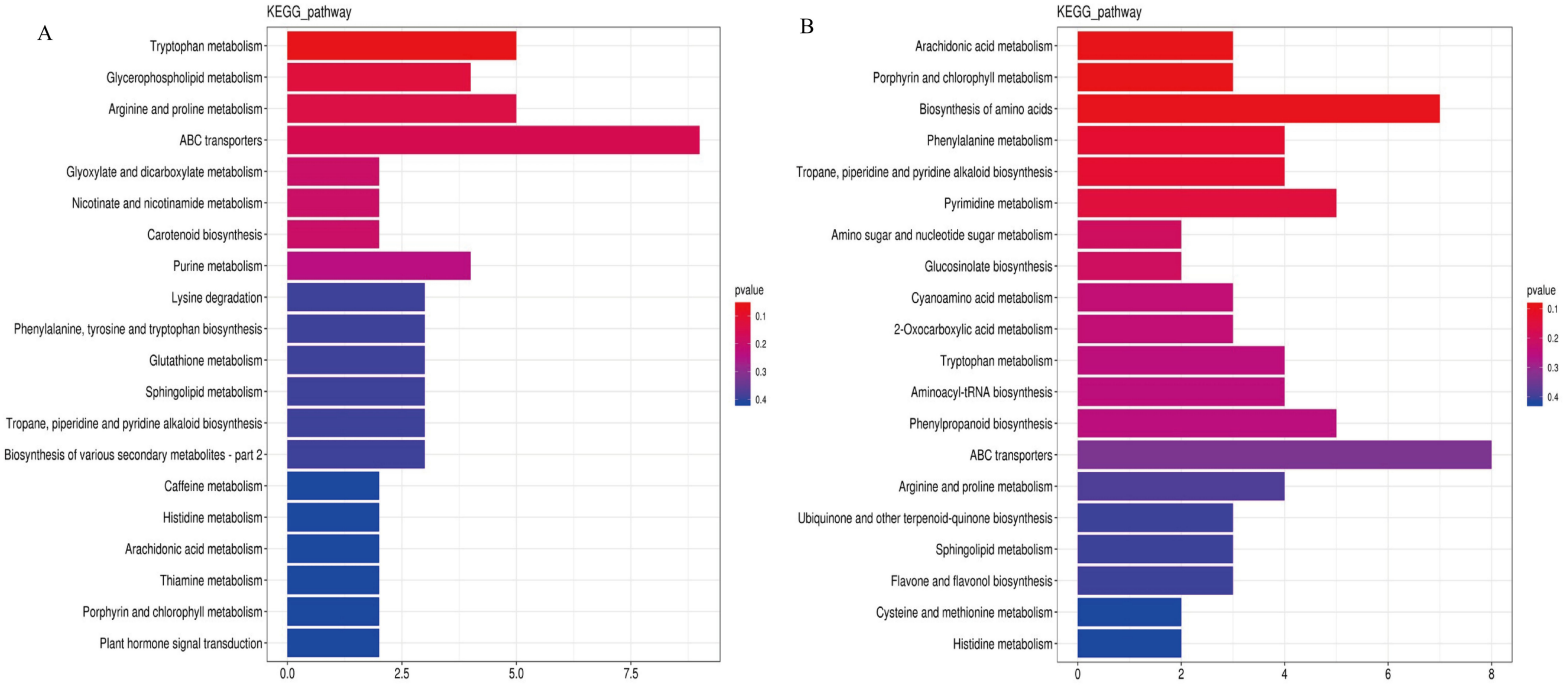
Enrichment analysis of DMs KEGG metabolic pathway. **(A)**: Late tetrad; **(B)**: Early mononuclear stage.

The DMs were enriched and analyzed based on metabolic pathways, and the late tetrad and early monocytes were clustered (**Figure 8**). The results showed that the tetrad stage was enriched in energy, phenylpropane, flavonoid, and glucose metabolism at the later stage. The early mononuclear stage showed enrichment in lipid, amino acid, and phenylpropane metabolism as well as other pathways.

### 3.10 Correlation and pathway co-enrichment analysis of DEGs and DMs

**Figure 9:**
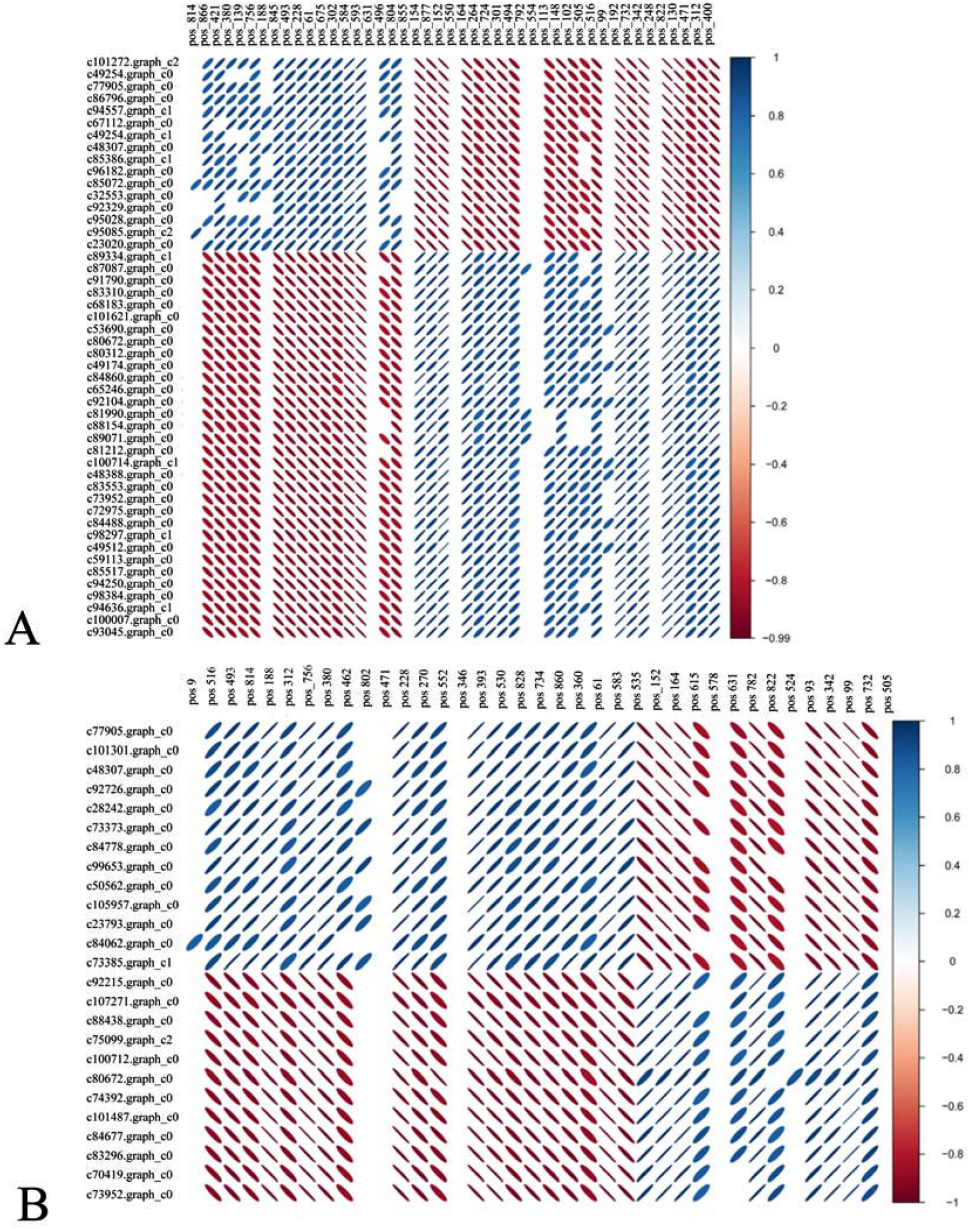
**(A)** represents the tetrad stage while, **(B)** represents the early mononuclear stage. Top DMs (top) and Top DEGs (left) correlation heatmap. Blue represents positive correlation, red represents negative correlation, the flatter the oval, the higher the absolute value of the correlation, only the combination of the statistical test of correlation *P*<0.05 is shown.

**Figure 10:**
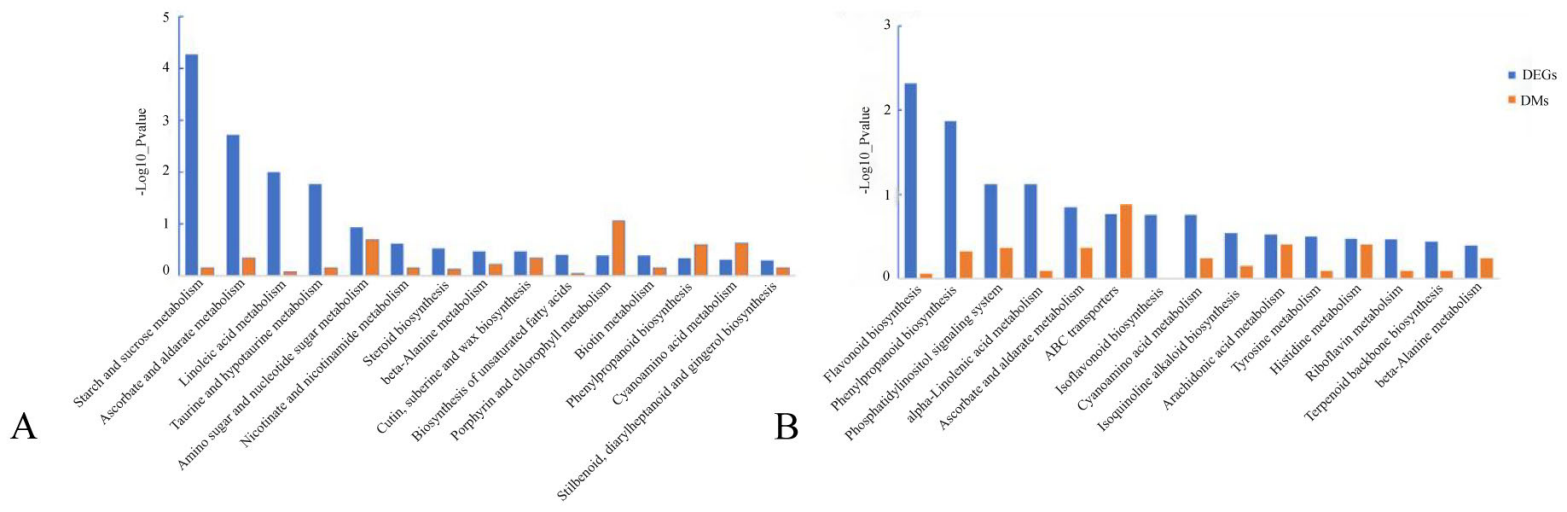
Statistical map of DEGs and DMs KEGG co-enrichment pathway. **(A)**: late anther tetrads, **(B)**: early mononuclear.

**Figure 11:**
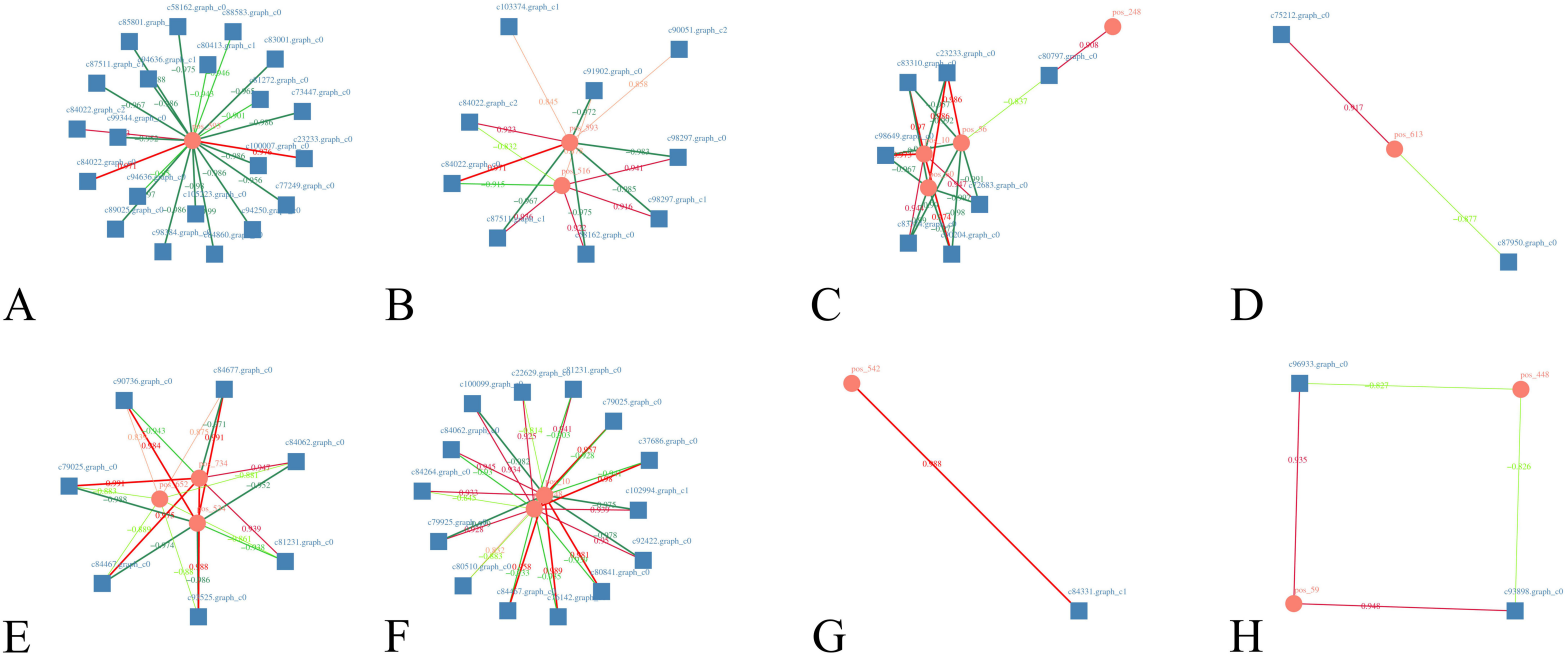
Co-expression network diagram of DEGs and DMs. **(A–D)**: late anther tetrads, **(E–H)**: early mononuclear; Squares represent genes, circles represent metabolites, red positive correlation, green negative correlation, the darker the color, the greater the absolute value of the correlation; **(A)**: Starch and sucrose metabolism, **(B)**: Amino sugar and nucleotide sugar metabolism, **(C)**: Phenylpropanoid biosynthesis, **(D)**: Biosynthesis of unsaturated fatty acids, **(E)**: Flavonoid biosynthesis, **(F)**: Isoflavonoid biosynthesis, **(G)**: Arachidonic acid metabolism, **(H)**: Isoquinoline alkaloid biosynthesis.

DEGs and DMs with significant differences were selected using R language corrplot package. Based on Pearson correlation coefficient, the correlation was calculated between DEGs and DMs (**Figure 9**). KEGG enrichment was performed on DEGs and DMs and a common pathway was obtained. The results showed that (**Figure 10**) the top ranking pathways at the late tetrad stage were starch and sucrose metabolism, ascorbic acid and uronic acid metabolism, linoleic acid metabolism, taurine and low taurine metabolism, amino acid sugar and nucleotide sugar metabolism, niacin and niacinamide metabolism, steroid biosynthesis, β-alanine metabolism, keratin, folinic acid and wax biosynthesis, and unsaturated fatty acid biosynthesis. The top early mononuclear pathways were flavonoid biosynthesis, phenylpropionate biosynthesis, phosphatidylinolenic acid signaling system, α-linolenic acid metabolism, ascorbic acid and uronic acid metabolism, ABC transport vehicles, isoflavone biosynthesis, cyanuric acid metabolism, isoquinoline alkaloid biosynthesis, and arachidonic acid metabolism. Many metabolic pathways, such as sucrose and starch, phenylpropyl, and isoflavones, involved in anther development were reported. At the two early stages of anther development, four metabolic pathways were selected and Cytoscape software was used to plot the network as shown in (**Figure 11**). It was observed that one metabolite was regulated by one or more genes, among which phenylpropane, isoflavone, and flavonoid metabolism were regulated by different genes to different extent.

### 3.11 QRT-PCR validation of DEGs

**Figure 12:**
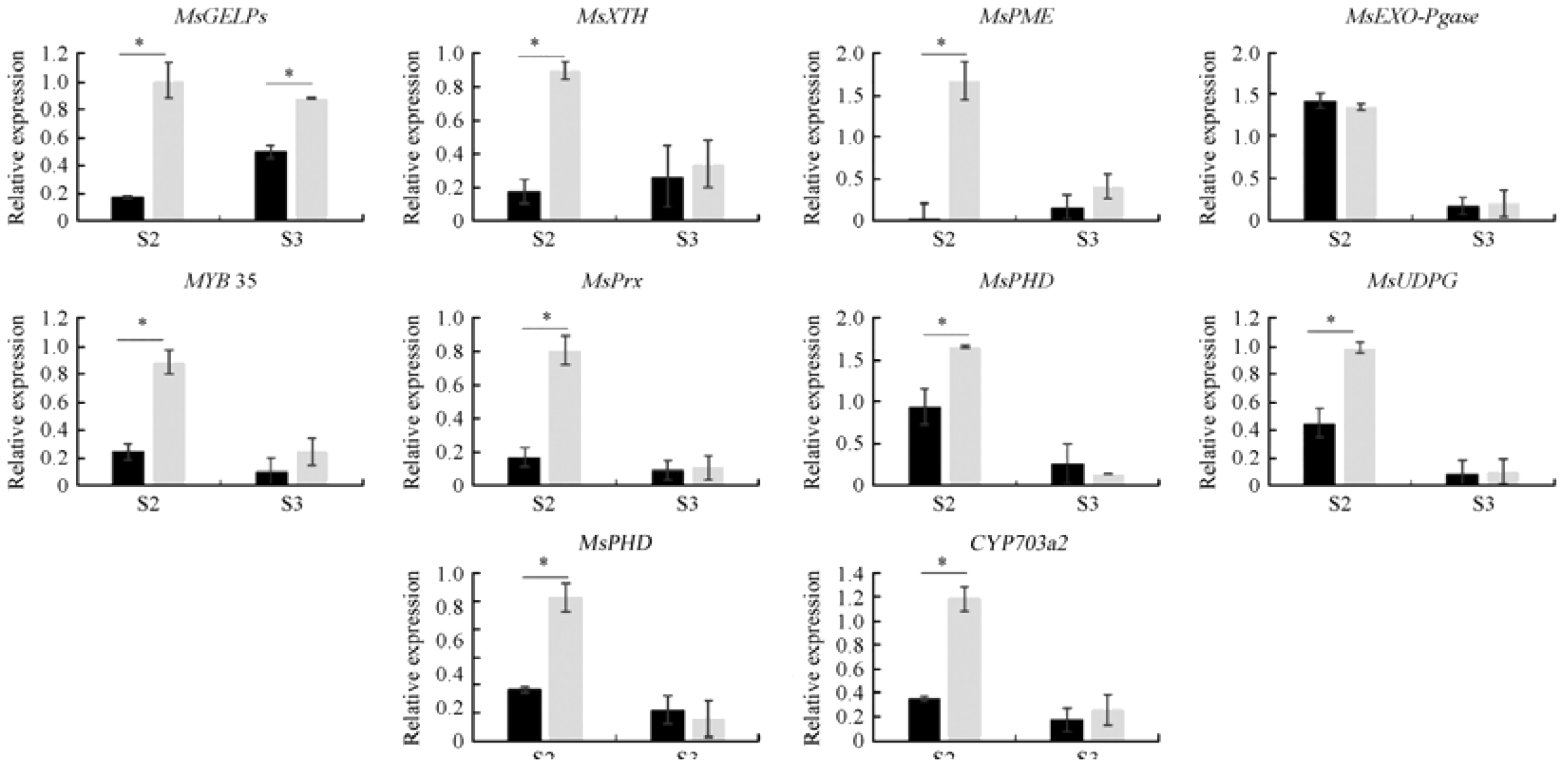
Expression pattern analysis of 10 candidate genes. * Indicates a significant difference between those two lines at the 0.05 level.

To verify the results of transcriptome and metabolome sequencing, 10 DEGs upstream of DMs were selected from different metabolic pathways. The expression patterns observed in metabolome sequencing were similar to those obtained by transcriptome sequencing (**Figure 12**). However, the expression of some genes (exogalacturonase) did not vary significantly between the two lines, owing to the low expression of this gene or the poor specificity of qRT-PCR primer. Primers are shown in **Supplementary Table 7**. *MsGDSL*, *MYB35*, *MsXTH*, *MsPME*, *C4H*, *MsPrx*, *MsPHD*, *CYP703a2*, and *MsUGPD* were downregulated during anther development, indicating that DMs are regulated by upstream DEGs.

### 3.12 Detection of physiological indicators

**Figure 13:**
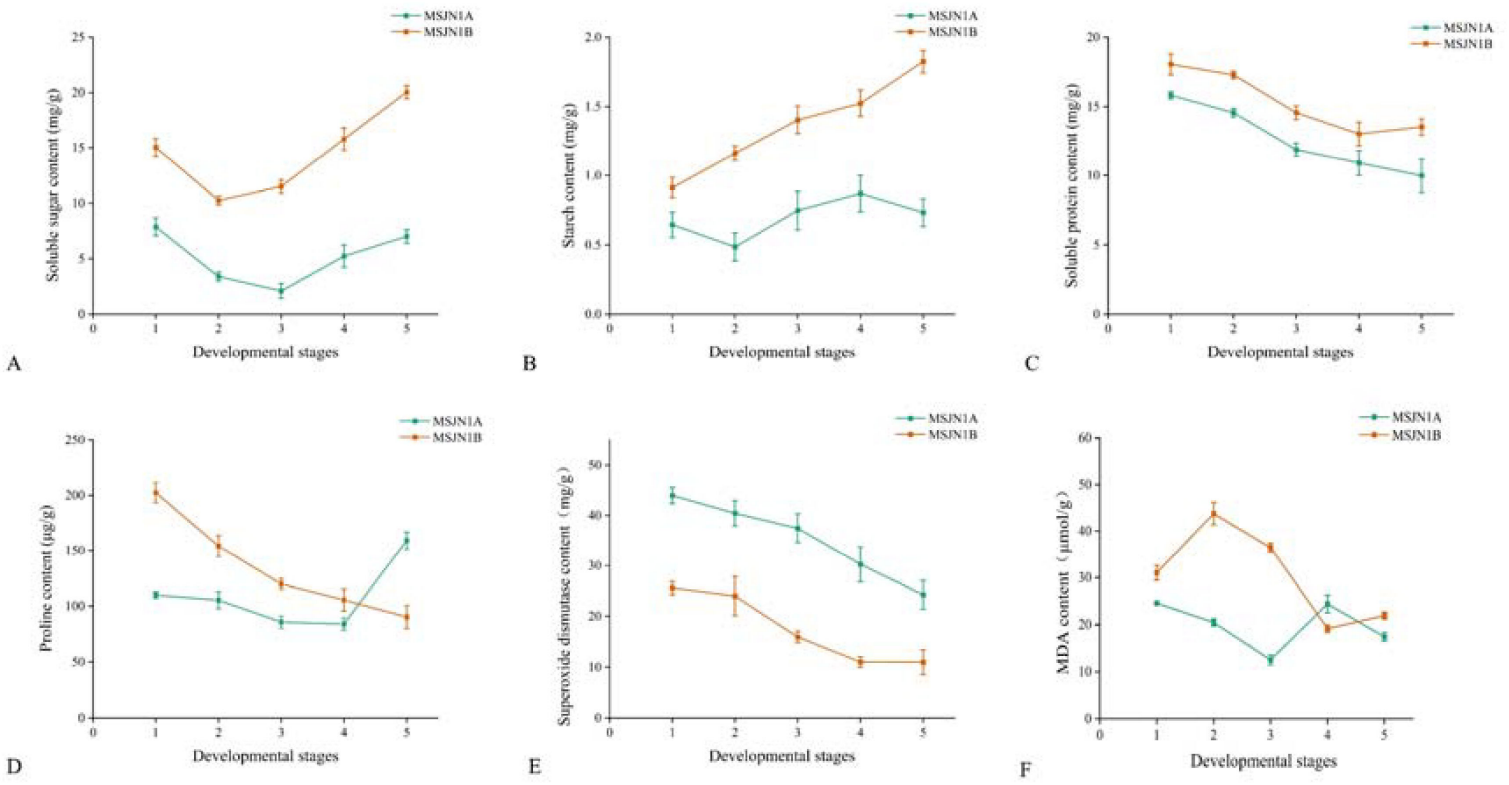
Determination of flower bud physiological indexes of sterile line MS-GN1A and maintainer line MS-GN1B at different developmental stages. **(A)**: Soluble sugar; **(B)**: starch; **(C)**: Soluble protein; **(D)**: proline; **(E)**: Superoxide dismutase; **(F)**: malondialdehyde.

Plant anthers undergo a series of complex physiological and biochemical processes during development, and these changes may directly and indirectly affect anther abortion. At the bud stage, 3~5 sterile buds and branches of the upper inflorescence of the maintainer buds were randomly selected and divided into five stages according to their morphological characteristics, microscopic examination, and development time. Then, the buds at different development stages were tested. The contents of soluble sugar (SS), starch (Starch), soluble protein (SP), free proline (PRO), SOD, and MDA in the sterile (MSJN1A) and maintainer lines (MSJN1B) at five different stages of anther development were measured. During flower bud development and pollen maturing, the contents of SS, Starch, SP, and PRO in the flower buds of the sterile line MS-GN1A showed a deficiency at each developing stage (**Figure 13**). The proline contents of the sterile (MS-GN1A) and maintainer lines (MS-GN1B) were significantly different at each developmental stage. The proline content of the maintainer line at the first four developmental stages was higher than that of the sterile line, and the proline content of the sterile line at the last stage was higher than that of the maintainer line. The deficiency and sudden increase in proline content during flower bud development may affect changes in metabolic substance structure and metabolic imbalance and lead to microspore sterility and abortion. The activities of MDA and SOD were higher in MS-GN1A than those in MS-GN1B. Compared with the cytoplasmic male sterility line and its maintainer line, nutrient deficiency is the main cause of male sterility and abortion during flower development.

## 4 Discussion

### 4.1 Abnormal degradation of tapetum cells causes anther microspore abortion

In this study, anthers from the early stage of anther development were selected for generating semi-thin sections. The results showed that the tapetum layer of the sterile line was not degraded relative to that of the maintainer line, and the inner wall of the anther was thicker. At the mononuclear microspore formation stage, the pollen grains were irregular in shape and dispersed in the anther sac in a crumpled state. In addition, some pollen grains were degraded, and the tapetum layer of the sterile line was not completely degraded during this process. This morphology of CMS anthers was similar to other higher plants, such as wheat (*Triticum aestivum* L.) (Ge et al., 2023) and pepper (*Capsicum annuum* L.) (Qiu et al., 2018). It was also consistent with Wang et al. findings who reported that abnormal degradation of the tapetum layer led to alfalfa anther abortion (Wang et al., 2013). No obvious vacuolar structure was observed in the center of microspores in the mononuclear sterile line. Studies have shown that pollen fertility was closely related to the presence of dynamic changes in the central large vacuole (Aouali et al., 2001). After degradation, nutrients and water are stored here while maintaining pollen morphology, osmotic pressure, and tolerance to changes in the external environment (Ugalde et al., 2016; Zhang et al., 2018). Therefore, it was speculated that the expressions of genes involved in apoptosis in the anthers of sterile lines were inhibited. The tapered layer did not degrade normally during the tetrad stage, which resulted in the change in the outer pollen wall and the disappearance of the central vacuole. Due to high energy requirement during pollen development and the production of a large number of reactive oxygen species, the lack of outer pollen wall and vacuole regulation, and microspore membrane peroxidation eventually leads to pollen abortion in MSJN1A.

### 4.2 Multiple metabolic pathway disorders lead to MSJN1A pollen abortion

During anther development, the tapetum layer—the raw material “warehouse” during pollen grain development—gradually degenerates after the tetrad stage (Han et al., 2022). Timely degradation of tapetum cells and callose decomposition provide enzymes and a large number of substrates for outer pollen wall formation (Guo and Liu, 2012). These substrates synthesize sporopolynes under the action of various enzymes and are transported to the inner cavity of the pollen and deposited into the outer wall of the pollen, which provides a barrier for anther development. Therefore, the entire process of anther development is interlinked, and the abnormality of any link will lead to pollen abortion (Lallemand et al., 2013). The supply of various nutrients, such as insoluble polysaccharides, lipids, and proteins, was increased to meet the changes in anther structure development. In this process, the metabolism of fatty acids, organic acids, amines, amino acids, sugars, polyols, and nucleotides are interrelated and participate in the TCA cycle and glycolysis, while providing energy needed for anther and pollen development (Tang et al., 2018). Sucrose transporters play a pivotal regulatory role in the development of anthers in alfalfa. By facilitating the efficient transport and distribution of sucrose (Sameeullah et al., 2013), these transporters support essential processes such as energy metabolism, cell wall remodeling, and the physiological stability of anthers and pollen (Lemoine, 2000). Furthermore, sucrose transporters may work synergistically with several key genes involved in anther. Development, including *MYB35* and *MsGDSL*. *MYB35* is a vital transcription factor that governs multiple stages of pollen and microspore development. The regulation of sucrose metabolism by sucrose transporters may influence *MYB35* expression, subsequently affecting the expression of downstream pollen-specific genes, which ultimately impacts pollen maturation and quality (Ito et al., 2004). Meanwhile, GDSL Esterases/Lipases are closely involved in cell wall remodeling and pollen tube growth. The carbon provided by sucrose transporters likely contributes directly to the synthesis and degradation of these cell wall components. *MsGDSL* proteins may modulate cell wall flexibility and degradation, promoting pollen tube growth and extension within the anther (Yan et al., 2024). Xu et al. reported through transcriptomic analysis that differentially expressed transcription factor genes (MYB4 and bHLH18) exhibited significantly reduced expression in the sterile line, confirming the importance of transcription factors in male sterility. In Arabidopsis (Xu et al., 2022), TDF1/MYB35 regulates anther structural formation and tapetal development (Zhu et al., 2008), while in rice, OsbHLH138 is identified as a key regulatory gene for thermo-sensitive male sterility (Cheng et al., 2020). Therefore, the interaction between sucrose transporters and these genes is crucial for the proper development of anthers and pollen in alfalfa. Metabolome sequencing analysis showed that there were significant abnormalities in sucrose and starch metabolism, phenylpropane metabolism, amino acid metabolism, lipid metabolism, and flavonoid metabolism in anthers of sterile lines during early anther development. Transcriptome and qRT-PCR analyses showed that multiple upstream transcription factors of the *MsGELPs* gene, such as MYB 35 and PHD finger protein male sterility 1, were downregulated in the sterile line. MYB 35 is known to participate in the biosynthesis of jasmonic acid, auxin, playing a crucial role in anther wall formation and anther dehiscence (Jeena et al., 2021). Yang et al. demonstrated that, in a male-sterile mutant of rice (TIP3), the PHD finger protein regulates tapetal cell degradation and Ubisch body formation (Yang et al., 2019b). Additionally, zinc finger proteins are closely associated with vacuole formation and phosphatidylinositol metabolism regulation. Based on these results, we hypothesize that the downregulation of MYB 35 and PHD finger protein male sterility 1 leads to decreased expression of the *MsGELPs* gene in the sterile line, resulting in abnormal tapetal cell degradation, defective pollen wall structure, and abnormal central vacuole formation in microspores, ultimately causing microspore abortion.

Among them, proline has strong physiological activity, which can provide energy, ammonia source, and carbon skeleton for pollen germination and pollen tube extension and is used for protein synthesis. The change of its content was proportional to the fertility of pollen (Wang et al., 2018). Therefore, the change of flower bud proline content can be used as one of the indices of alfalfa pollen fertility. Kiran et al. showed that the carbohydrate and proline metabolism disorder in anthers at low temperature led to sterility of chickpea flower powder (Kiran et al., 2021). In this study, a large number of amino acid deficiencies were detected by GC-MS, and amino acids, including proline, aspartic acid, histidine, and leucine were significantly decreased, which was consistent with the content of free proline and SP during early anther development of the sterile line. The results were similar to the physiological indices of the CMS line of carrot (Đurić et al., 2024). Therefore, the decrease in proline content was one of the possible causes of anther abortion in sterile lines.

Carbohydrate metabolism is one of the basic metabolic pathways of plant growth and development (Kretschmer et al., 2017). It is not only a substrate for energy metabolism but also an important part of the plant cell wall. During anther development, starch stored in tapetal cells is degraded into sucrose and transported to anther granules, and then starch is synthesized again under the action of UDP-glucose dehydrogenase and stored in sporoids. We found that the anther content of ADP-glucose—the direct raw material for starch synthesis—was higher in the sterile line than in the maintainer line, whereas the expression of starch synthesis *MsUGPD* (c58162.graph_c0) was lower in the maintainer line. This indicated that starch granules in the tapetum cells of the sterile line were degraded to a certain extent. However, due to the lack of subsequent reuse sites, the content of ADP-glucose was increased, which hindered the transfer of starch in the tapetum to microsporites and could not provide adequate nutrition for subsequent anther development. This result was consistent with the anther starch deficiency of the sterile line relative to the maintainer line.

Phenylpropane metabolism has a variety of metabolic branches and its downstream metabolites, such as flavonoids, lignin, and cinnamic acid amide play an important role in anther development (Xue et al., 2020; Dong and Lin, 2021). Lignin can be deposited during anther development, forming lignin layers that increase the hardness and stability of anthers (Liu et al., 2018). In Arabidopsis c4h mutants, plant growth and lignin accumulation were inhibited, apical dominance was lost, and male sterility was observed (Schilmiller et al., 2009). Thevenin et al. induced sterility and nanosomia in Arabidopsis by inhibiting two enzymes CCR and CAD involved in the lignin biosynthesis (Thévenin et al., 2011). Flavonoids are the most diverse metabolites in phenylpropane metabolic pathway, and flavonols are involved in maintaining pollen fertility (Daryanavard et al., 2023). *AtMYB21* and *AtMYC2* have been reported (Liu et al., 2021) to interact and involved in regulating stamen development and seed production in *Arabidopsis* (Qi et al., 2015). Similarly, we found abnormal phenylpropane metabolism and its downstream flavonoid metabolic pathway during early anther development of sterile lines with more DEGs (*CYP703a2*, *MsC4H*, and *MsMTD*) downregulated and more DMs (L-phenylalanine, coumarin, trans-cinnamic acid, and n15/10-triferuloyl spermidine) enriched in this pathway. Among them, *CYP703a2* was a cytochrome gene specifically involved in pollen development, which mainly catalyzed the inner chain hydroxylation of C-7 position of medium chain saturated fatty acids, thus affecting the synthesis of pollen ectosporine (Morant et al., 2003). This might be related to the late formation of outer pollen wall in the tetrad or the formation of abnormal structure and components.

The formation and homeostasis of the anther cuticle and pollen wall are regulated by lipid metabolism (Liu et al., 2022). Anther cuticle is composed of keratin and wax, which is a mixture of alkanes, olefins, fatty alcohols, and ultra-long chain fatty acids (Tian et al., 2017). Hydroxyl and epoxy long chain fatty acids are synthesized into keratin monomer under the action of acyltransferase and then polymerized into keratin (Gómez et al., 2015). Zafar et al. showed that abnormal fatty acid metabolism and Reactive Oxygen Species (ROS) imbalance resulted in significantly reduced amounts of corneous wax and keratins in anthers and decreased fertility of mutant rice (Zafar et al., 2020). ROS play a crucial regulatory role in anther and tapetum development, primarily by promoting programmed cell death (PCD) in the tapetum, modulating the anther developmental process, and maintaining cellular homeostasis. Firstly, ROS can promote PCD in tapetal cells, ensuring timely degeneration of the tapetum to provide essential nutritional support for pollen development. If the tapetum does not degenerate at the appropriate time, pollen development may be adversely affected (Yi et al., 2016). Secondly, ROS function as signaling molecules that regulate different stages of anther development. In cotton anther development, ROS act as key signaling molecules that regulate tapetal PCD and developmental progression. By being released from mitochondria and transmitted to the nucleus, ROS trigger normal tapetum development and the transition to a secretory state, ensuring anther maturation. However, in CMS lines, decreased ROS-scavenging capacity leads to ROS accumulation, resulting in tapetal abnormalities and ultimately causing pollen abortion (Yang et al., 2018). Finally, maintaining ROS homeostasis is crucial for preventing cellular damage, especially in cases of CMS, where ROS accumulation can lead to abnormal anther and tapetum development, ultimately causing pollen abortion. Thus, through the regulation of PCD, signal transduction, and homeostasis maintenance, ROS play an essential role in the normal development of anthers and tapetum (Balk and Leaver, 2001). The outer pollen wall is mainly formed by the deposition of sporopolynes, which is composed of various long chain fatty acids and phenols (Jiang et al., 2012). Wang et al. have shown that transcription factors, such as AMS and MS 188, can activate the expression of spore pollen biosynthesis pathway and synthesize sporopolynes to rapidly form pollen walls (Wang et al., 2018). Metabolome and transcriptome analysis showed that lipid metabolism was abnormal in sterile lines during anther development with abnormal expression of various lipid metabolites. In this study, the contents of long chain fatty acids (palmitic acid) and medium chain fatty acids (canoleic acid) in sterile lines anthers decreased, whereas those of short chain fatty acids (propionic acid) increased. The blockage of extension of fatty acid carbon chain and abnormal raw materials for sporopoline synthesis might lead to anther cragging and pollen microspore abortion.

## Data Availability

The data presented in this study are deposited in the Zenodo repository, accession number https://doi.org/10.5281/zenodo.13635670.
