# Supplementary material for: Transcriptomics and metabolomics analyses reveal pollen abortion mechanism in alfalfa early stage male sterile lines
Source: Front Plant Sci. 2024 Dec 17;15:1464747. doi: 10.3389/fpls.2024.1464747 (PMC11687225; doi:10.3389/fpls.2024.1464747)
Supplement: Supplementary file 1 [file DataSheet1.docx]

Supplementary Material

Transcriptomics and metabolomics analyses reveal pollen abortion mechanism in alfalfa early stage male sterile lines

Huicai Cai ^1#^, Shuhe Zhang ^1#^, Weijie Yu ^1^, Xue Jia ^1^, Lan Yu ^1^, Bo Xu ^1*^, Yingzhe Wang ^2*^

*** Correspondence:** Bo Xu: [xubo6299@jlau.edu.cn](mailto:xubo6299@jlau.edu.cn)

*** Correspondence:** Yingzhe Wang: [yingzhe120@163com](mailto:yingzhe120@163com)

**Table 1 Statistical table of transcript assembly results**

| Length range/bp | Transcript | | Unigene | |  |
| --- | --- | --- | --- | --- | --- |
|  |  |  |  |  |  |
|  | Number | Percentage | Number | Percentage |  |
| 200～300 | 44 483 | 18.78% | 33 682 | 33.60% |  |
| 300～500 | 45 480 | 19.20% | 25 352 | 25.29% |  |
| 500～1 000 | 54 598 | 23.05% | 17 807 | 17.76% |  |
| 1 000～2 000 | 55 489 | 23.43% | 13 349 | 13.32% |  |
| 2 000+ | 36 818 | 15.54% | 10 063 | 10.04% |  |
| Total number | 236 868 |  | 100 253 |  |  |
| Total length | 257 222 216 |  | 80 015 464 |  |  |
| N50 N50 length | 1 750 |  | 1 493 |  |  |
| Mean length | 1 085.93 |  | 798.14 |  |  |

**Table 2 Statistical table of the functional genes annotated**

| Anther developmental stage | Late anther tetrads stage | Early mononuclear stage |  |
| --- | --- | --- | --- |
|  |  |  |  |
| Annotated numbers | 1 622 | 1 761 |  |
| COG | 351 | 386 |  |
| GO | 906 | 975 |  |
| KEGG | 391 | 430 |  |
| KOG | 714 | 773 |  |
| Pfam | 1 004 | 1 060 |  |
| Swiss-Prot | 960 | 975 |  |
| eggNOG | 1 193 | 1 262 |  |
| Nr | 1 607 | 1 727 |  |

**Table 3 TOP DEGs Function Annotation of late anther tetrads**

| Sequence ID | log_2_FC | Zhongmu No.1 ID | Function annotation |  |
| --- | --- | --- | --- | --- |
|  |  |  |  |  |
|  |  |  |  |  |
| c101272.graph_c2 | 7.77 | MsG0780039618.01.T01 | Protein processing in endoplasmic reticulum (ko04141) |  |
| c49254.graph_c0 | 6.27 | MsG0580024574.01.T01 | DNA replication (ko03030) |  |
|  |  |  | Nucleotide excision repair (ko03420) |  |
|  |  |  | Mismatch repair (ko03430) |  |
|  |  |  | Homologous recombination (ko03440) |  |
| c77905.graph_c0 | 5.88 | Not found | Purine metabolism (ko00230) |  |
|  |  |  | Pyrimidine metabolism (ko00240) |  |
|  |  |  | RNA polymerase (ko03020) |  |
| c86796.graph_c0 | 5.65 | MsG0480021617.01.T01 | Plant-pathogen interaction (ko04626) |  |
| c94557.graph_c1 | 5.31 | MsG0480022581.01.T01 | Steroid biosynthesis (ko00100) |  |
|  |  |  | Sesquiterpenoid and triterpenoid biosynthesis (ko00909) |  |
|  |  |  |  |  |
| c67112.graph_c0 | 5.26 | MsG0380014878.01.T01 | Oxidative phosphorylation (ko00190) |  |
| c49254.graph_c1 | 5.21 | MsG0580024574.01.T01 | DNA replication (ko03030) |  |
|  |  |  | Nucleotide excision repair (ko03420) |  |
|  |  |  | Mismatch repair (ko03430) |  |
|  |  |  | Homologous recombination (ko03440) |  |
| c48307.graph_c0 | 4.91 | Not found | DNA replication (ko03030) |  |
|  |  |  | Nucleotide excision repair (ko03420) |  |
|  |  |  | Mismatch repair (ko03430) |  |
|  |  |  | Homologous recombination (ko03440) |  |
| c85386.graph_c1 | 4.82 | MsG0580029116.01.T01 | DNA replication (ko03030) |  |
|  |  |  | Nucleotide excision repair (ko03420) |  |
|  |  |  | Mismatch repair (ko03430) |  |
|  |  |  | Homologous recombination (ko03440) |  |
| c96182.graph_c0 | 4.49 | MsG0180000170.01.T01 | DNA replication (ko03030) |  |
|  |  |  | Nucleotide excision repair (ko03420) |  |
|  |  |  | Mismatch repair (ko03430) |  |
|  |  |  | Homologous recombination (ko03440) |  |
| c85072.graph_c0 | 4.41 | MsG0780040755.01.T01 | Endocytosis (ko04144) |  |
| c32553.graph_c0 | 4.37 | MsG0180003746.01.T01 | mRNA surveillance pathway (ko03015) |  |
| c92329.graph_c0 | 4.34 | MsG0580024320.01.T01 | DNA replication (ko03030) |  |
|  |  |  | Nucleotide excision repair (ko03420) |  |
|  |  |  | Mismatch repair (ko03430) |  |
|  |  |  | Homologous recombination (ko03440) |  |
| c95028.graph_c0 | 4.18 | MsG0880043336.01.T01 | DNA replication (ko03030) |  |
|  |  |  | Nucleotide excision repair (ko03420) |  |
|  |  |  | Mismatch repair (ko03430) |  |
|  |  |  | Homologous recombination (ko03440) |  |
| c95085.graph_c2 | 4.07 | MsG0180003703.01.T01 | Ribosome biogenesis in eukaryotes (ko03008) |  |
| c23020.graph_c0 | 4.02 | MsG0480018099.01.T01 | RNA transport (ko03013) |  |
| c89334.graph_c1 | -4.18 | MsG0480022342.01.T01 | Protein processing in endoplasmic reticulum (ko04141) |  |
| c87087.graph_c0 | -4.26 | MsG0180002285.01.T01 | Glycerolipid metabolism (ko00561) |  |
|  |  |  | Glycerophospholipid metabolism (ko00564) |  |
| c91790.graph_c0 | -4.32 | MsG0780041813.01.T01 | Oxidative phosphorylation (ko00190) |  |
| c83310.graph_c0 | -4.34 | MsG0180000119.01.T01 | Phenylpropanoid biosynthesis (ko00940) |  |
| c68183.graph_c0 | -4.36 | MsG0180005882.01.T01 | Oxidative phosphorylation (ko00190) |  |
| c101621.graph_c0 | -4.41 | MsG0180005340.01.T01 | RNA transport (ko03013) |  |
|  |  |  | mRNA surveillance pathway (ko03015) |  |
|  |  |  | RNA degradation (ko03018) |  |
| c53690.graph_c0 | -4.45 | MsG0580024151.01.T01 | Ribosome (ko03010) |  |
| c80672.graph_c0 | -4.49 | Not found | Glutathione metabolism (ko00480) |  |
| c80312.graph_c0 | -4.59 | MsG0580028095.01.T01 | Alanine, aspartate and glutamate metabolism (ko00250) |  |
|  |  |  |  |  |
|  |  |  | beta-Alanine metabolism (ko00410) |  |
|  |  |  | Taurine and hypotaurine metabolism (ko00430) |  |
|  |  |  | Butanoate metabolism (ko00650) |  |
| c49174.graph_c0 | -4.68 | MsG0580028095.01.T01 | DNA replication (ko03030) |  |
|  |  |  | Nucleotide excision repair (ko03420) |  |
|  |  |  | Mismatch repair (ko03430) |  |
|  |  |  | Homologous recombination (ko03440) |  |
| c84860.graph_c0 | -4.69 | MsG0880047070.01.T01 |  |  |
|  |  |  | Pentose and glucuronate interconversions (ko00040) |  |
|  |  |  | Starch and sucrose metabolism (ko00500) |  |
| c65246.graph_c0 | -4.72 | MsG0580028983.01.T01 | Plant-pathogen interaction (ko04626) |  |
| c92104.graph_c0 | -4.82 | MsG0280010151.01.T01 | Glycolysis/Gluconeogenesis (ko00010) |  |
|  |  |  | Carbon fixation in photosynthetic organisms (ko00710) |  |
|  |  |  | Carbon metabolism (ko01200) |  |
|  |  |  | Biosynthesis of amino acids (ko01230) |  |
| c81990.graph_c0 | -4.97 | MsG0780036132.01.T01 | Oxidative phosphorylation (ko00190) |  |
|  |  |  | Phagosome (ko04145) |  |
| c88154.graph_c0 | -4.99 | MsG0480020618.01.T01 | Purine metabolism (ko00230) |  |
|  |  |  | Pyrimidine metabolism (ko00240) |  |
|  |  |  | RNA polymerase (ko03020) |  |
| c89071.graph_c0 | -5.19 | MsG0480021446.01.T01 | Oxidative phosphorylation (ko00190) |  |
|  |  |  | Phagosome (ko04145) |  |
| c81212.graph_c0 | -5.39 | MsG0180004198.01.T01 | Protein processing in endoplasmic reticulum (ko04141) |  |
| c100714.graph_c1 | -5.39 | MsG0280007405.01.T01 | Plant-pathogen interaction (ko04626) |  |
| c48388.graph_c0 | -5.43 | MsG0480022888.01.T01 | Plant-pathogen interaction (ko04626) |  |
| c83553.graph_c0 | -5.61 | MsG0780039166.01.T01 | Ribosome biogenesis in eukaryotes (ko03008) |  |
| c73952.graph_c0 | -5.76 | MsG0480022872.01.T01 | DNA replication (ko03030) |  |
|  |  |  | Nucleotide excision repair (ko03420) |  |
|  |  |  | Mismatch repair (ko03430) |  |
|  |  |  | Homologous recombination (ko03440) |  |
| c72975.graph_c0 | -5.77 | Not found | Nitrogen metabolism (ko00910) |  |
| c84488.graph_c0 | -5.83 | Not found | Nucleotide excision repair (ko03420) |  |
|  |  |  | Ubiquitin mediated proteolysis (ko04120) |  |
| c98297.graph_c1 | -6.13 | MsG0280010749.01.T03 | Amino sugar and nucleotide sugar metabolism (ko00520) |  |
| c49512.graph_c0 | -6.28 | MsG0580028280.01.T02 | Ubiquitin mediated proteolysis (ko04120) |  |
| c59113.graph_c0 | -6.44 | MsG0480022221.01.T01 | Ascorbate and aldarate metabolism (ko00053) |  |
| c85517.graph_c0 | -6.52 | MsG0180004113.01.T01 | Protein processing in endoplasmic reticulum (ko04141) |  |
| c94250.graph_c0 | -6.81 | MsG0280009277.01.T01 | Pentose and glucuronate interconversions (ko00040) |  |
|  |  |  | Starch and sucrose metabolism (ko00500) |  |
| c98384.graph_c0 | -7.19 | MsG0880047069.01.T01 | Pentose and glucuronate interconversions (ko00040) |  |
|  |  |  | Starch and sucrose metabolism (ko00500) |  |
| c94636.graph_c1 | -7.37 | MsG0280007949.01.T01 | Pentose and glucuronate interconversions (ko00040) |  |
|  |  |  | Starch and sucrose metabolism (ko00500) |  |
| c100007.graph_c0 | -7.58 | MsG0780041411.01.T02 | Pentose and glucuronate interconversions (ko00040) |  |
|  |  |  | Starch and sucrose metabolism (ko00500) |  |
| c93045.graph_c0 | -7.71 | MsG0680030722.01.T01 | Pentose and glucuronate interconversions (ko00040) |  |

**Table 4 TOP DEGs function annotation of early mononuclear**

| Sequence ID | log2FC | Zhongmu No.1 ID | Function annotation |  |
| --- | --- | --- | --- | --- |
|  |  |  |  |  |
| c77905.graph_c0 | 7.19 | Not found | Purine metabolism (ko00230) |  |
|  |  |  | Pyrimidine metabolism (ko00240) |  |
|  |  |  | RNA polymerase (ko03020) |  |
| c101301.graph_c0 | 5.71 | MsG0480020539.01.T01 | Plant hormone signal transduction (ko04075) |  |
| c48307.graph_c0 | 5.68 | Not found | DNA replication (ko03030) |  |
|  |  |  | Nucleotide excision repair (ko03420) |  |
|  |  |  | Mismatch repair (ko03430) |  |
|  |  |  | Homologous recombination (ko03440) |  |
| c92726.graph_c0 | 5.32 | MsG0280008959.01.T01 | DNA replication (ko03030) |  |
|  |  |  | Nucleotide excision repair (ko03420) |  |
|  |  |  | Mismatch repair (ko03430) |  |
|  |  |  | Homologous recombination (ko03440) |  |
| c28242.graph_c0 | 5.03 | Not found | Oxidative phosphorylation (ko00190) |  |
| c73373.graph_c0 | 4.86 | Not found | Oxidative phosphorylation (ko00190) |  |
| c84778.graph_c0 | 4.66 | MsG0780037601.01.T01 | DNA replication (ko03030) |  |
|  |  |  | Nucleotide excision repair (ko03420) |  |
|  |  |  | Mismatch repair (ko03430) |  |
|  |  |  | Homologous recombination (ko03440) |  |
| c99653.graph_c1 | 4.56 | MsG0780037601.01.T01 | DNA replication (ko03030) |  |
|  |  |  | Nucleotide excision repair (ko03420) |  |
|  |  |  | Mismatch repair (ko03430) |  |
|  |  |  | Homologous recombination (ko03440) |  |
| c50562.graph_c0 | 4.46 | Not found | Oxidative phosphorylation (ko00190) |  |
| c105957.graph_c0 | 4.38 | Not found | Phosphatidylinositol signaling system (ko04070) |  |
| c23793.graph_c0 | 4.13 | MsG0880043189.01.T01 | RNA degradation (ko03018) |  |
| c84062.graph_c0 | 4.08 | MsG0880045990.01.T01 | Phenylpropanoid biosynthesis (ko00940) |  |
|  |  |  | Flavonoid biosynthesis (ko00941) |  |
|  |  |  | Stilbenoid, diarylheptanoid and gingerol biosynthesis (ko00945) |  |
|  |  |  |  |  |
| c73385.graph_c1 | 4.02 | Not found | RNA transport (ko03013) |  |
| c92215.graph_c0 | -4.02 | MsG0380017320.01.T01 | Starch and sucrose metabolism (ko00500) |  |
| c107271.graph_c0 | -4.02 | MsG0280008333.01.T01 | Spliceosome (ko03040) |  |
|  |  |  | Protein processing in endoplasmic reticulum (ko04141) |  |
|  |  |  | Endocytosis (ko04144) |  |
| c88438.graph_c0 | -4.03 | MsG0480023389.01.T01 | Starch and sucrose metabolism (ko00500) |  |
| c75099.graph_c2 | -4.29 | MsG0380016245.01.T01 | Pentose and glucuronate interconversions (ko00040) |  |
| c100712.graph_c0 | -4.91 | MsG0580026317.01.T01 | Glycosaminoglycan degradation (ko00531) |  |
| c80672.graph_c0 | -4.92 | Not found | Glutathione metabolism (ko00480) |  |
| c74392.graph_c0 | -5.06 | MsG0880046424.01.T01 | Pentose and glucuronate interconversions (ko00040) |  |
| c101487.graph_c0 | -5.23 | MsG0680030946.01.T01 | DNA replication (ko03030) |  |
|  |  |  | Nucleotide excision repair (ko03420) |  |
|  |  |  | Mismatch repair (ko03430) |  |
|  |  |  | Homologous recombination (ko03440) |  |
| c84677.graph_c0 | -5.4 | MsG0380015575.01.T01 | Flavonoid biosynthesis (ko00941) |  |
| c83296.graph_c0 | -5.73 | Not found | Cysteine and methionine metabolism (ko00270) |  |
|  |  |  | Valine, leucine and isoleucine degradation (ko00280) |  |
|  |  |  | Valine, leucine and isoleucine biosynthesis (ko00290) |  |
|  |  |  | Pantothenate and CoA biosynthesis (ko00770) |  |
|  |  |  | 2-Oxocarboxylic acid metabolism (ko01210) |  |
|  |  |  | Biosynthesis of amino acids (ko01230) |  |
| c70419.graph_c0 | -5.98 | MsG0280009082.01.T01 | DNA replication (ko03030) |  |
|  |  |  | Nucleotide excision repair (ko03420) |  |
|  |  |  | Mismatch repair (ko03430) |  |
|  |  |  | Homologous recombination (ko03440) |  |
| c73952.graph_c0 | -6.36 | MsG0480022872.01.T01 | DNA replication (ko03030) |  |
|  |  |  | Nucleotide excision repair (ko03420) |  |
|  |  |  | Mismatch repair (ko03430) |  |
|  |  |  | Homologous recombination (ko03440) |  |

**Table 5 DMs of late anther tetrads**

| Metabolite ID | Metabolite name | log2FC | Pvalue | VIP | regulated |
| --- | --- | --- | --- | --- | --- |
| pos_814 | Naringenin-7-O-Glucoside | 7.13 | 3. 15E-03 | 1.39 | up |
| pos_866 | Ginkgetin | 6.87 | 1.24E-02 | 1.26 | up |
| pos_421 | 2-Amino-1-phenylethanol | 5. 14 | 6.58E-04 | 1.48 | up |
| pos_380 | 3-Isochromanone | 4.97 | 9.71E-04 | 1.47 | up |
| pos_ 139 | Delphinidin | 4.81 | 1.55E-02 | 1.21 | up |
|  | 3-(6-p-coumaroylgalactoside) |  |  |  |  |
| pos_756 | Cytosine | 4.31 | 4.54E-03 | 1.36 | up |
| pos_ 188 | Anibine | 4.22 | 1.23E-03 | 1.43 | up |
| pos_845 | Sulfamethoxypyridazine | 3.92 | 5.31E-04 | 1.46 | up |
| pos_493 | Prolyl-Glutamate | 3.73 | 2.91E-04 | 1.51 | up |
| pos_228 | 1,4,5-Naphthalenetriol | 3.41 | 9.78E-05 | 1.55 | up |
| pos_61 | 5,7,8,2',4'-Pentahydroxyisoflavon e | 2.61 | 1.70E-03 | 1.41 | up |
| pos_675 | O-Succinyl-L-homoserine | 2.58 | 1.08E-04 | 1.5 | up |
| pos_302 | Aminoparathion | 2.49 | 4.45E-02 | 1.03 | up |
| pos_584 | Camptothecin | 2.39 | 5.89E-06 | 1.57 | up |
| pos_593 | ADP-glucose | 2.27 | 8.33E-09 | 1.59 | up |
| pos_631 | Deoxyuridine monophosphate (dUMP) | 2. 14 | 1.44E-02 | 1. 14 | up |
| pos_496 | Lecanoric acid | 2.07 | 9.58E-04 | 1.42 | up |
| pos_804 | Sissotrin | 2.06 | 1.63E-04 | 1.44 | up |
| pos_855 | Estrone 3-glucuronide | 2.03 | 5.42E-03 | 1.28 | up |
| pos_ 154 | Met His Val Phe | -2.15 | 8. 18E-08 | 1.58 | down |
| pos_877 | L-Cysteinesulfinic acid | -2.31 | 2.97E-05 | 1.56 | down |
| pos_ 152 | Methyl methacrylate | -2.32 | 6.80E-05 | 1.56 | down |
| pos_550 | Astragalin | -2.43 | 3. 15E-02 | 1.08 | down |
|  | 4-[2-(5-Carboxy-2-hydroxy-3-me |  |  |  |  |
| pos_ 164 | thoxyphenyl)- | -2.48 | 2.59E-05 | 1.57 | down |
|  | 2-oxoethylidene]-2-hydroxy-2-pe ntenedioate |  |  |  |  |
|  | Apigenin |  |  |  |  |
|  | 7-[feruloyl-(->2)-glucuronyl- ( 1->2)-glucuronide] |  |  |  | down |
| pos_264 | 4'-glucuronide | -2.57 | 1.29E-03 | 1.36 |  |
| pos_724 | Leukotriene D4 | -2.58 | 2.80E-06 | 1.56 | down |
| pos_301 | Apigenin 4'-[feruloyl-(->2)- | -2.6 | 3.35E-07 | 1.57 | down |
|  | glucuronyl-(1->2)-glucuronide] |  |  |  |  |
| pos_494 | Serinyl-Aspartate | -2.7 | 1.08E-02 | 1.22 | down |
| pos_792 | Aspartyl-Tyrosine | -2.86 | 3. 17E-02 | 1.08 | down |
| pos_554 | Inosine 5'-monophosphate (IMP) | -2.89 | 4.94E-02 | 1.01 | down |
| pos_ 113 | Luteolin 7,3',4'-triglucuronide | -2.89 | 4.44E-04 | 1.44 | down |
| pos_ 148 | Galangin | -3.02 | 2.93E-05 | 1.5 | down |
| pos_ 102 | Apigenin 7-[glucuronyl-(1->2)- glucuronide] 4'-glucuronide | -3. 12 | 5.21E-04 | 1.41 | down |
| pos_505 | Theobromine | -3.13 | 9.78E-04 | 1.47 | down |
| pos_516 | UDP-D-Galactose | -3.13 | 2.76E-03 | 1.35 | down |
| pos_99 | 2-Dehydro-O-desmethylangolens in | -3.23 | 3. 10E-02 | 1.1 | down |
| pos_ 192 | Asp His Leu His | -3.27 | 5.88E-09 | 1.6 | down |
| pos_732 | 2-Furancarboxylic acid | -3.32 | 2.32E-05 | 1.57 | down |
| pos_342 | 2-Furanmethanol | -3.33 | 3.55E-05 | 1.56 | down |
| pos_248 | N1,N5,N10-Triferuloyl spermidine | -3.37 | 1.59E-02 | 1.21 | down |
| pos_822 | D-Glucuronolactone | -3.76 | 3.00E-06 | 1.59 | down |
| pos_ 130 | Malvidin | -3.9 | 7.31E-08 | 1.6 | down |
|  | 3-glucoside-4-vinylphenol |  |  |  |  |
| pos_471 | Coproporphyrin I | -4.66 | 2.58E-03 | 1.39 | down |
| pos_312 | Asp Trp Arg Tyr | -5.08 | 9.45E-04 | 1.46 | down |
| pos_400 | 2,4-Diaminobutyric acid | -5.18 | 2. 17E-04 | 1.53 | down |

**Table 6 DMs of early mononuclear**

| Metabolite ID | Metabolite name | log2FC | Pvalue | VIP | regulated |
| --- | --- | --- | --- | --- | --- |
| pos_9 | N-Acetyl-L-aspartic acid | 12.48 | 4.49E-02 | 1.07 | up |
| pos_516 | UDP-D-Galactose | 4.81 | 2.59E-03 | 1.39 | up |
| pos_493 | Prolyl-Glutamate | 4.78 | 3.55E-03 | 1.37 | up |
| pos_814 | Naringenin-7-O-Glucoside | 4.66 | 7.86E-04 | 1.45 | up |
| pos_ 188 | Anibine | 4.55 | 1.80E-04 | 1.52 | up |
| pos_312 | Asp Trp Arg Tyr | 4.3 | 4.07E-03 | 1.38 | up |
| pos_756 | Cytosine | 4.23 | 2.64E-05 | 1.55 | up |
| pos_380 | 3-Isochromanone | 3.8 | 1.53E-05 | 1.54 | up |
| pos_462 | Cytidine 2',3'-cyclic phosphate | 3.52 | 1.24E-02 | 1.2 | up |
| pos_802 | Mangiferin | 3.51 | 3.46E-02 | 1.05 | up |
| pos_471 | Coproporphyrin I | 3.48 | 1.00E-02 | 1.3 | up |
| pos_228 | 1,4,5-Naphthalenetriol Quercetin | 3.48 | 7.72E-05 | 1.53 | up |
| pos_270 | 3-(6''-malonylglucoside)-7-rha mnoside | 3.39 | 4.75E-02 | 1.02 | up |
| pos_552 | Cycloheximide | 2.89 | 2.20E-05 | 1.48 | up |
| pos_346 | 4'-DEMETHYLEPIPODOPH YLLOTOXIN | 2.74 | 8. 11E-03 | 1.22 | up |
| pos_393 | Glutathione, oxidized | 2.47 | 2. 13E-06 | 1.57 | up |
| pos_530 | Parathion | 2.38 | 4.59E-05 | 1.52 | up |
| pos_828 | Pyridoxal (Vitamin B6) | 2.36 | 4.46E-05 | 1.55 | up |
| pos_734 | Neohesperidin | 2.27 | 4.36E-05 | 1.49 | up |
| pos_860 | 16, 16-dimethyl-PGA1 | 2.27 | 6.85E-04 | 1.44 | up |
| pos_360 | Eremopetasitenin C3 | 2.14 | 1.02E-04 | 1.51 | up |
| pos_61 | 5,7,8,2',4'-Pentahydroxyisofla vone | 2.13 | 2.50E-02 | 1.1 | up |
| pos_583 | 2-thiouridine | 2.12 | 6.58E-09 | 1.58 | up |
| pos_535 | CDP-Ethanolamine | 2.03 | 6. 16E-06 | 1.51 | up |
| pos_ 152 | Methyl methacrylate | -2.16 | 1.82E-04 | 1.51 | down |
|  | 4-[2-(5-Carboxy-2-hydroxy-3 |  |  |  |  |
| pos_ 164 | -methoxyphenyl)- | -2.32 | 6. 16E-05 | 1.53 | down |
|  | 2-oxoethylidene]-2-hydroxy-2 -pentenedioate |  |  |  |  |
| pos_615 | Methylephedrine | -2.43 | 1.03E-04 | 1.41 | down |
| pos_578 | Glucosaminic acid | -2.48 | 3.71E-04 | 1.47 | down |
| pos_631 | Deoxyuridine monophosphate (dUMP) | -2.52 | 4.37E-02 | 1.01 | down |
| pos_782 | 8-Hydroxy-2'-deoxyguanosine | -3.08 | 2.82E-03 | 1.36 | down |
| pos_822 | D-Glucuronolactone | -3.25 | 2.03E-04 | 1.52 | down |
| pos_524 | 5-methoxyuridine | -3.35 | 2.98E-02 | 1.09 | down |
| pos_93 | Pro Cys Ser Tyr | -4.14 | 1.03E-02 | 1.26 | down |
| pos_342 | 2-Furanmethanol | -4.27 | 1.42E-04 | 1.52 | down |
| pos_99 | 2-Dehydro-O-desmethylangol ensin | -4.49 | 1.34E-07 | 1.58 | down |
| pos_732 | 2-Furancarboxylic acid | -4.52 | 9.89E-05 | 1.55 | down |
| pos_505 | Theobromine | -4.76 | 4.36E-03 | 1.36 | down |

**Table 7 Primers used in the qRT-PCR**

| Gene ID | Accession ID | Gene name | Primer name | Primer sequence(5′-3′) |  |
| --- | --- | --- | --- | --- | --- |
|  |  |  |  |  |  |
|  |  |  |  |  |  |
| c89437.graph_c0 | OQ439928 | GDSL type esterase/lipase MsGELPs | MsGELPs-F | GCTCTAGAATGAATTCCATGAGGTTAATTCATGTAC |  |
|  |  |  | MsGELPs-R | CGAGCTCTTATAAACATGCCTTCCCAATTGG |  |
| c81458.graph_c0 | OQ439929 | Endoxyglucosylase hydrolase | MsXTH-F | TGGAGAAGCATGGGCAACACAAG |  |
|  |  | *MsXTH* | MsXTH-R | TTGGAACGCAAGCATTGGCATTG |  |
| c100007.graph_c0 | OQ439930 | Pectin methylase | MsPME-F | GGTTACCGCTGTTGCCCTTGAC |  |
|  |  | *MsPME* | MsPME-R | AAGACGACGGGAAGCAGGTTTAAC |  |
| c85801.graph_c0 | OQ439931 | Exopolygalacturonase | MsEXO-Pgase-F | GTGCTTCCCTCCCTGCTTCTTTG |  |
|  |  | *MsEXO*-*Pgase* | MsEXO-Pgase-R | CGGAGCTGTTATGGTGAGGTTAGTG |  |
| c78022.graph_c0 | OQ439932 | Cytochrome P450 | CYP703a2-F | AGCAATCAACGGATGAACCTTAGGC |  |
|  |  | *CYP703a2* | CYP703a2-R | TGATGGCTTTGGCTCGACTGTTTC |  |
| c104620.graph_c1 | OQ439933 | Cinnamic acid 4-hydroxylase | C_4_H-F | GCTTGTTCACGAGGCGATGTTATTG |  |
|  |  | *C*_4_*H* | C_4_H-R | CCTGAAGATCAAACGGGCGTAGC |  |
| c78766.graph_c0 | OQ439934 | peroxidase | MsPrx-F | GTGGAGGACCTAACTGGGAGCTAC |  |
|  |  | *MsPrx* | MsPrx-R | AGAGACCTTCAACAGTGGCATTTGG |  |
| c58162.graph_c0 | OQ439935 | Udp-glucose dehydrogenase | MsUGPD-F | ACCGAGTTGTTGCGTCCATGTTC |  |
|  |  | *MsUGPD* | MsUGPD-R | CCTTGTATCGCCTGTGTCCTTCTTG |  |
| c89108.graph_c0 | OQ439936 | PHD-finger type transcription factor | PHD-F | GCATTGGTTCCTTCAGCACAAGC |  |
|  |  | *MsPHD* | PHD -R | ACCCGTGTTGCCATTTCGATCC |  |
| c91687.graph_c0 | OQ439937 | MYB type transcription factor 35 | MYB 35-F | TGTTGCCCTTATCTCACTGTCCTTG |  |
|  |  | *MYB35* | MYB 35-R | GGTGGTTCAAGTGAAGGTTTTGAGG |  |
|  |  | β-actin(Internal reference) | β-actin-F | TGAGTGGTGGTACGACTATGTTCCC |  |
|  |  |  | β-actin-R | TCATGCTACTTGGTGCCAATGCTG |  |


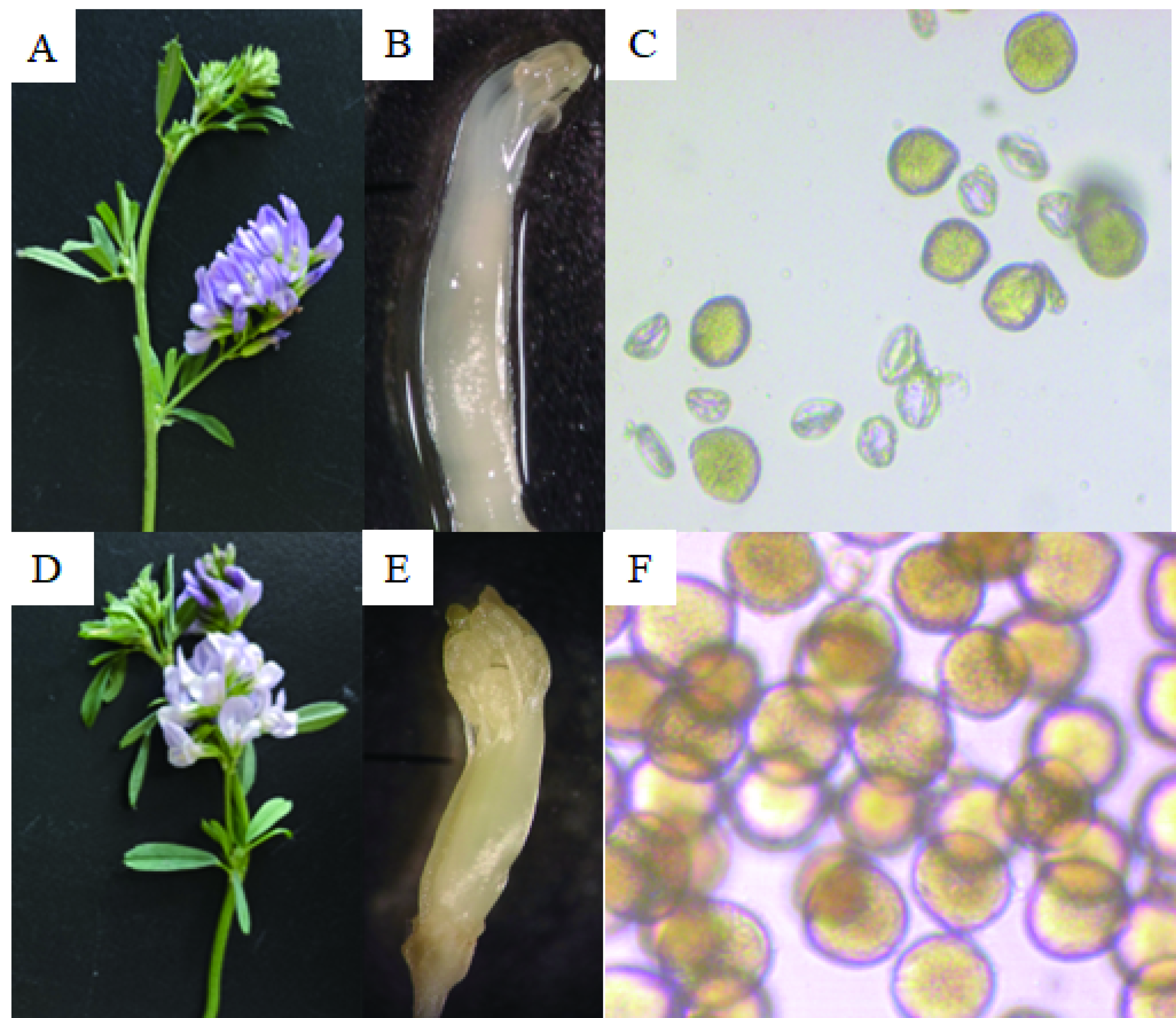


**Supplementary Figure 1.** Cytoplasmic male sterile lines and maintainer lines of alfalfa (*Medicago sativa* L.); (**A-C**) are sterile lines plants, stamens, and pollen, respectively. (**D-F**) are maintainer lines plants, stamens, and pollen, respectively.


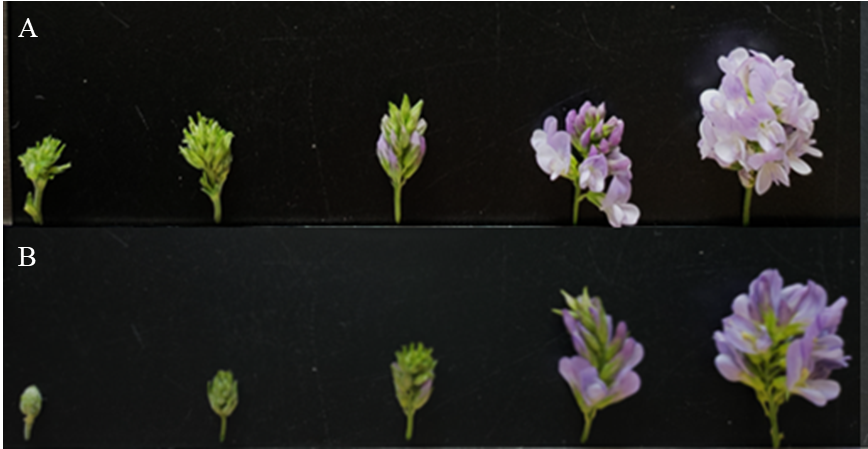


**Supplementary Figure 2.** (**A**) (MSJN1A) and (**B**) (MSJN1B) are the five periods of anther development from left to right, respectively.


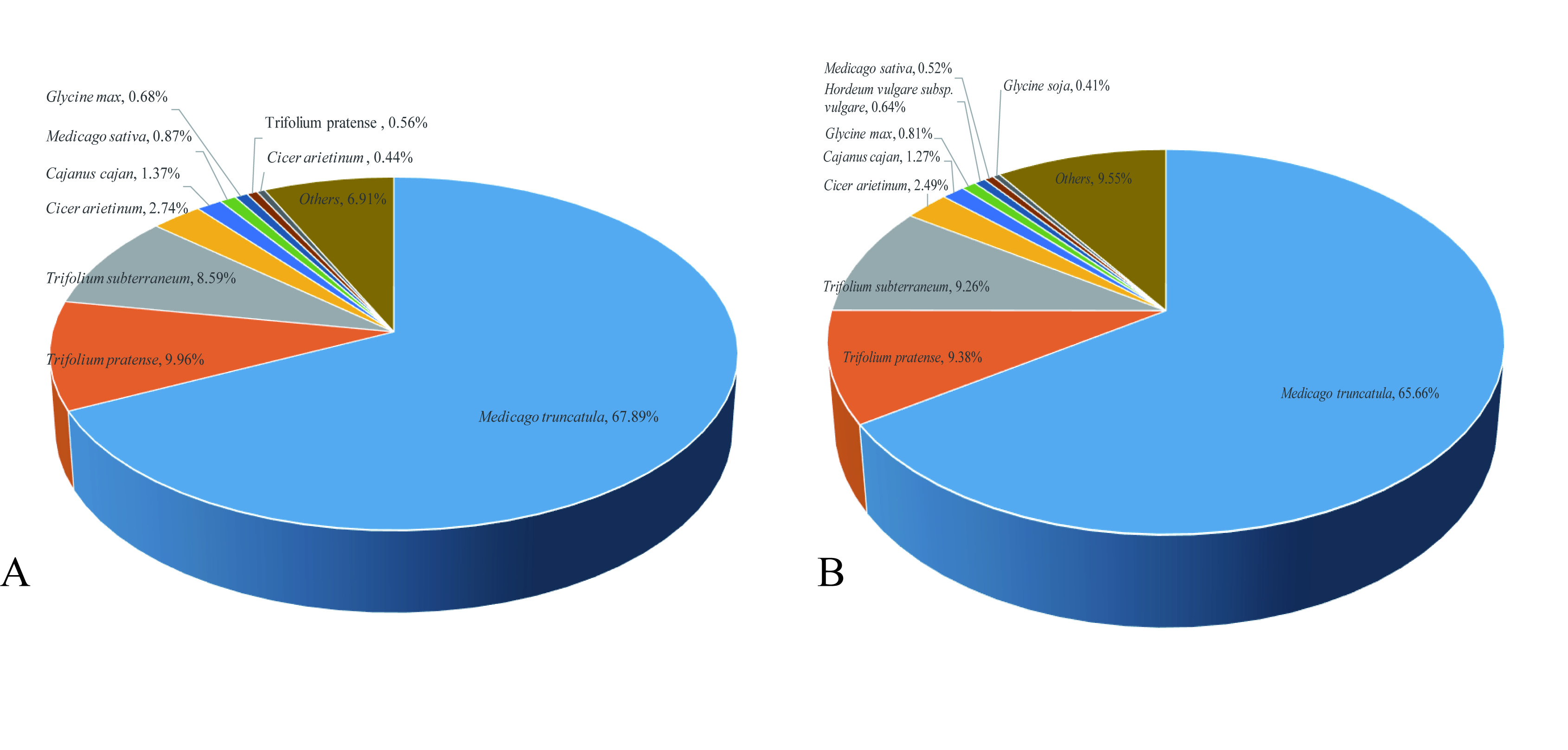


**Supplementary Figure 3.** (**A**) (MSJN1A) and (**B**) (MSJN1B) Nr function annotation for unigenes.


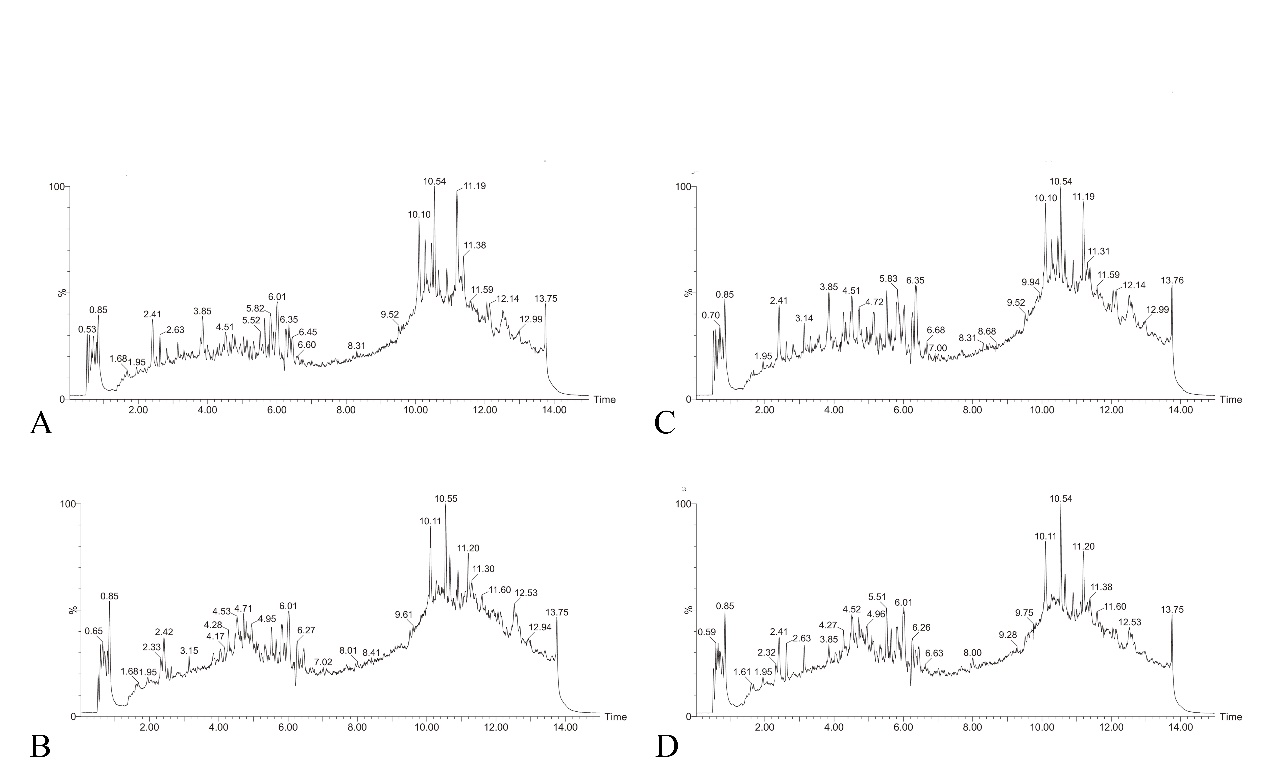
**Supplementary Figure 4.** Chromatogram of late tetrad and early mononuclear anthers of sterile and maintenance. (A): late sterile line tetrad, (B): early mononuclear stage of sterile line, (C): late retention line tetrad, (D): early retention line mononuclear.


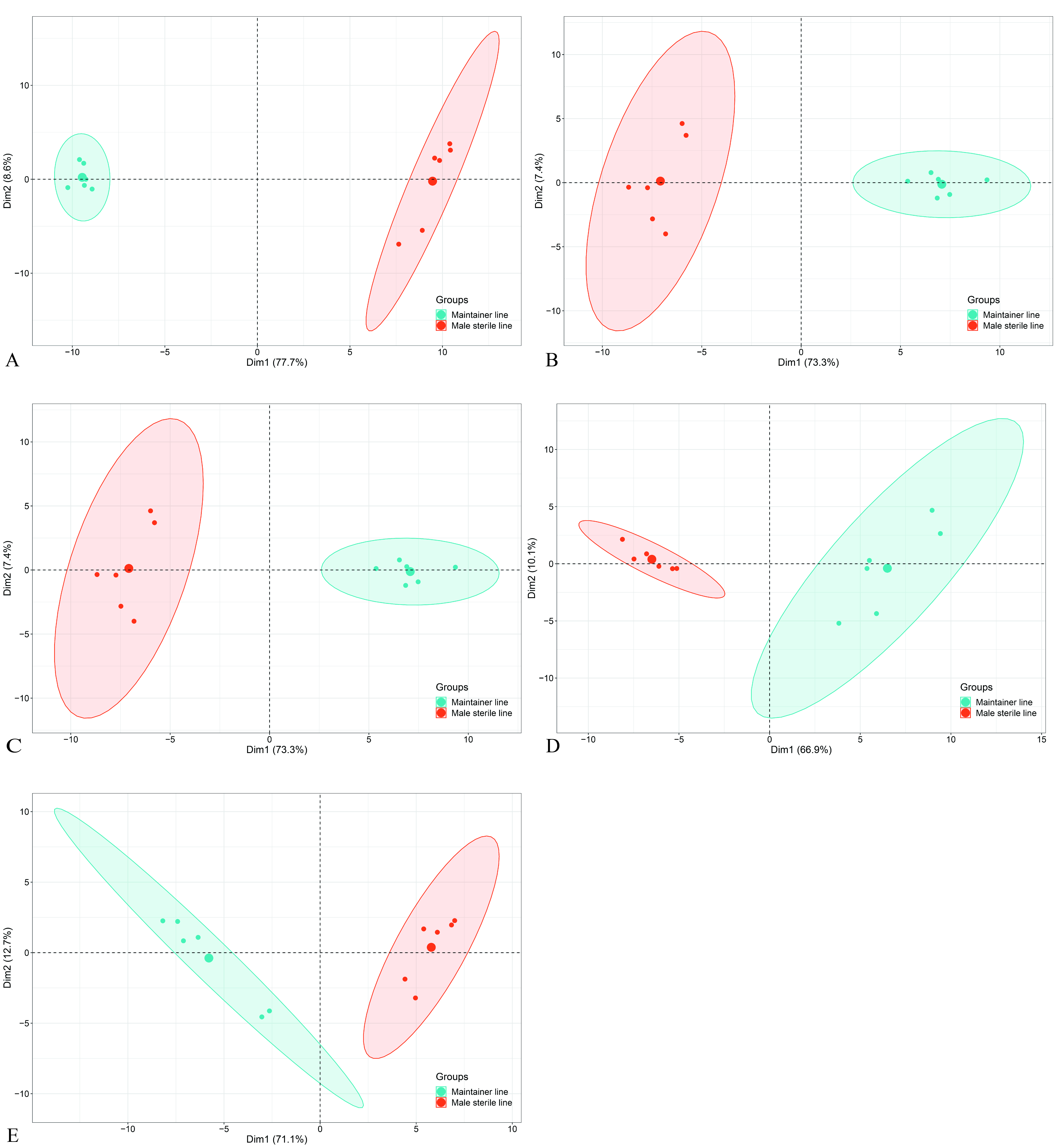


**Supplementary Figure 5.** PCA. (**A**): Late tetrad; (**B**): Early mononuclear stage; (**C**): Monokaryon stage; (**D**): Dikaryon stage; (**E**): Mature stage
